# Effect of Empagliflozin on the plasma lipidome in patients with type 2 diabetes mellitus: results from the EmDia clinical trial

**DOI:** 10.1186/s12933-025-02916-0

**Published:** 2025-09-08

**Authors:** Katrin I. Bauer, Dhanwin Baker, Raissa Lerner, Thomas Koeck, Gregor Buch, Zlatka Fischer, Robin Martens, Ekaterina E. Esenkova, Maximilian Nuber, Miguel A. Andrade-Navarro, Vincent ten Cate, Stefan Tenzer, Philipp S. Wild, Laura Bindila, Elisa Araldi

**Affiliations:** 1 Computational Biomedicine, Center for Thrombosis and Hemostasis (CTH), Mainz, Germany; 2 Clinical Epidemiology and Systems Medicine, Center for Thrombosis and Hemostasis (CTH), Mainz, Germany; 3 German Center for Cardiovascular Research (DZHK), Partner Site RhineMain, Mainz, Germany; 4 Clinical Lipidomics Unit, Institute of Physiological Chemistry, University Medical Center, Mainz, Germany; 5 Institute of Medical Biostatistics, Epidemiology and Informatics (IMBEI), University Medical Center of the Johannes-Gutenberg University Mainz, Mainz, Germany; 6 Institute of Organismic and Molecular Evolution, Johannes-Gutenberg University Mainz, Mainz, Germany; 7 Center for Thrombosis and Hemostasis, University Medical Center of the Johannes Gutenberg-University Mainz, Mainz, Germany; 8 Institute of Immunology, University Medical Center of the Johannes-Gutenberg University Mainz, Mainz, Germany; 9 Preventive Cardiology and Preventive Medicine, Department of Cardiology, University Medical Center of the Johannes-Gutenberg University Mainz, Mainz, Germany; 10 Institute for Molecular Biology (IMB), Mainz, Germany; 11 Systems Medicine Laboratory, Department of Medicine and Surgery, University of Parma, Parma, Italy

**Keywords:** SGLT2i, HFpEF, Lipidomics, Empagliflozin, Clinical trial

## Abstract

**Background:**

Sodium-glucose cotransporter 2 (SGLT2) inhibitors, such as Empagliflozin, are antidiabetic drugs that reduce glucose levels and have emerged as a promising therapy for patients with heart failure (HF), although the exact molecular mechanisms underlying their cardioprotective effects remain to be fully elucidated. The EmDia study, a randomized, double-blind trial conducted at the University Medical Center of Mainz, has confirmed the beneficial effects of Empagliflozin in HF patients after both one and twelve weeks of treatment. In this work, we aimed to assess whether changes in lipid profiles driven by Empagliflozin use in HF patients in the EmDia trial could assist in gaining a better understanding of its cardioprotective mechanisms.

**Methods:**

Lipid analysis of blood plasma from 144 patients from the EmDia trial was conducted using 4D-LC-TIMS/IMS lipidomics. Lipid signatures after treatment for one and twelve weeks, respectively, were obtained with sparse group LASSO regularized regression models. Linear regression models were employed to highlight associations between significantly changed clinical traits and lipids.

**Results:**

The lipid signatures after one week of treatment consisted of 37 lipids from the lipid groups lysophosphatidylcholine (LPC), phosphatidylcholine (PC), phosphatidylethanolamine (PE), sphingomyelin (SM), and triacylglycerol (TG). After twelve weeks, the signature comprised 24 lipids from the same five lipid groups, along with Ceramides (Cer). Three of five lipids altered at both time points showed consistent directional trends. Empagliflozin treatment led to significant alterations in the lipidome, including increases in both beneficial lipids, such as LPCs, and potentially harmful species, notably ceramides, which have been implicated in lipotoxicity and cardiovascular risk.

**Conclusion:**

This study identified distinct lipid signatures associated with Empagliflozin treatment after both one and twelve weeks, respectively, with five lipids overlapping between signatures and three with consistent directions, revealing that some of the beneficial effects of Empagliflozin could be through lipid modulation. Notably, Empagliflozin-modulated lipids associated with changes in clinical traits and lipid-specific profiles among clinical subgroups were observed. However, challenges remain in establishing direct associations between individual lipids and clinical outcomes. Future research integrating lipidomics data with other omics datasets could provide a more comprehensive understanding of the identified lipid signatures and their potential roles in health and diseases.

**Trial registration:**

ClinicalTrials.gov; NCT02932436. Registration date, 2016/10/13.

**Graphical abstract:**

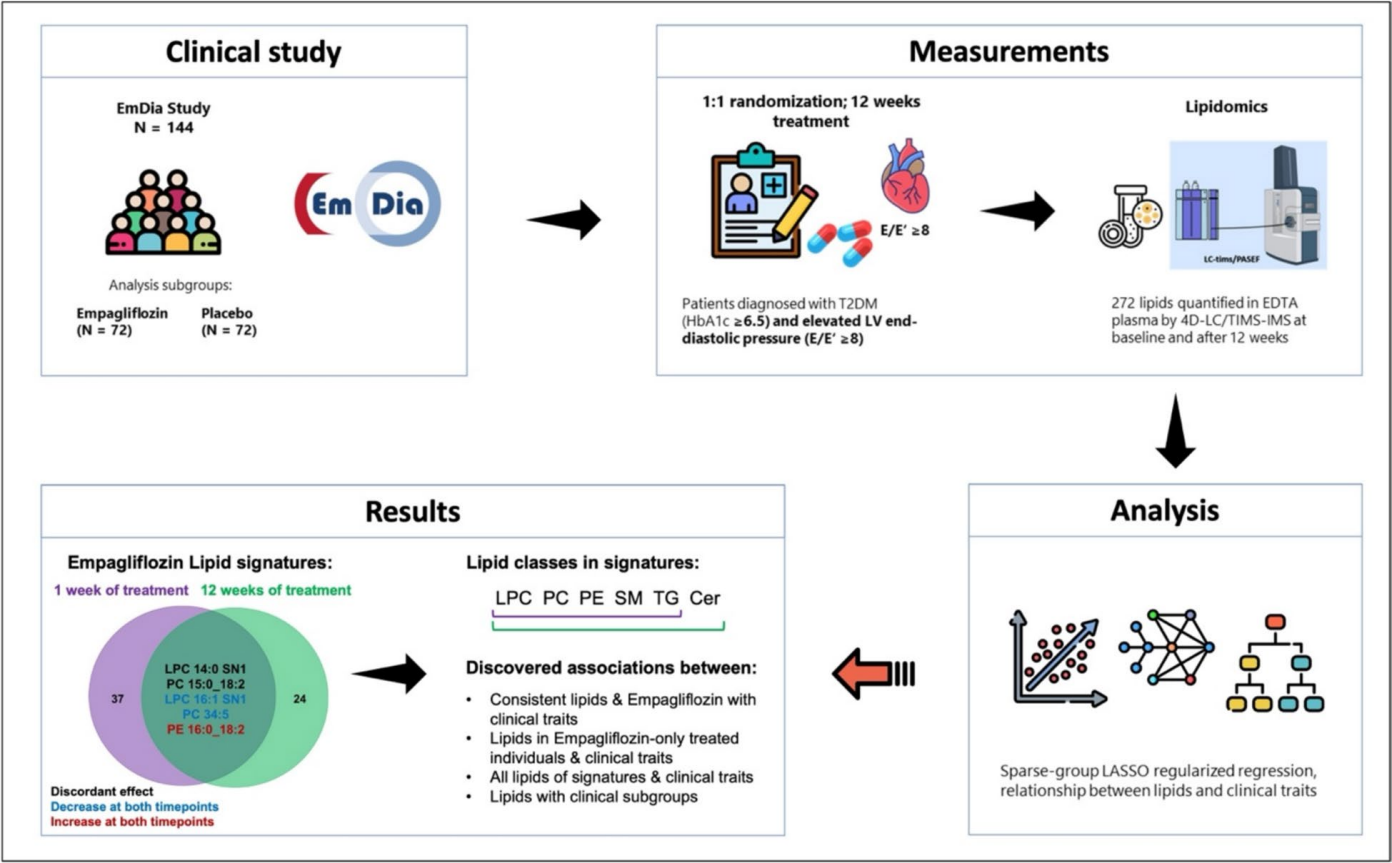

**Supplementary Information:**

The online version contains supplementary material available at 10.1186/s12933-025-02916-0.

## Research insights

**What is currently known about this topic?**

- SGLT2i reduce glucose concentration in serum by inducing glycosuria. Patients diagnosed with diabetes mellitus type 2 show a reduced risk of death from any cause after treatment with SGLT2i Empagliflozin, including death from cardiovascular causes, as well as decreased hospitalization due to heart failure. Further, SGLT2i are the first-line approved drug class for managing HFpEF.

**What is the key research question?**

- Can the analysis of lipidome changes due to Empagliflozin treatment help explain the largely unknown molecular mechanisms responsible for the cardioprotective effects that are not attributable to its well-established glucose excretion capabilities in heart failure?

**What is new?**

- Lipidomics is a rather recent research field and might help to understand biochemical mechanisms. While there have already been several clinical studies on the effects of Empagliflozin, none of them so far has been focused on exploring lipidome changes in the blood plasma of human individuals by using mass spectrometry, specifically 4D-LC/TIMS-IMS.

**How might this study influence clinical practice?**

- Lack of effective treatment for HFpEF makes it important to investigate the mechanisms behind Empagliflozin positive effects. Lipids might help explain this mechanism and can be both a diagnostic tool and a therapeutic target for HFpEF.

## Background

Complications derived from cardiovascular diseases (CVD), particularly from the heart failure syndrome (HF), are one of the leading causes of death worldwide [1]. A greater understanding of the aetiology of the syndrome, as well as a more detailed molecular characterization are needed for patient stratification and personalized therapies and have the potential to significantly increase the health and lifespan of individuals with HF. SGLT2 inhibitors (SGLT2i) are a class of antidiabetic drugs that reduce glucose concentration in serum by inducing glycosuria. Patients with type 2 diabetes mellitus treated with the SGLT2i Empagliflozin experienced a reduced risk of death from any cause, including death from cardiovascular causes, as well as decreased hospitalization due to HF compared to placebo [2–7]. For their cardioprotective effect, SGLT2i have emerged as promising therapies for HF, especially for HF with preserved ejection fraction (HFpEF) [8, 9]. In fact, SGLT2i are the first-line approved drug class for managing HFpEF [10].

The EmDia study is a single-center randomized double-blind controlled clinical trial designed to assess how SGLT2i benefits patients with HF. It combines a comprehensive clinical assessment and extensive molecular phenotyping to understand how Empagliflozin affects patients with type 2 diabetes mellitus (glycosylated hemoglobin, HbA1c ≥ 6.5%) and elevated left ventricular end-diastolic pressure (E/E’′≥ 8). In the EmDia study, in line with other similar studies [2, 11, 12], Empagliflozin showed a significant improvement of the left ventricular diastolic function after twelve weeks of therapy [7]. Additionally, a decrease in HbA1c, body mass index (BMI), uric acid, blood pressure, hematocrit, and mean cell hemoglobin concentration (MCHC) could be observed, as well as an increase of the red blood cell (RBC) count and hemoglobin [13].

While Empagliflozin has proven positive outcomes in HF [5, 14, 15], the molecular mechanisms responsible for the cardioprotective effects are not attributable to its well-established glucose excretion capabilities and are mostly unknown. This study sheds light on the lipidome changes due to Empagliflozin treatment and attempts to increase our understanding of Empagliflozin’s role in HF through changes in plasma lipid composition.

Lipid molecules are responsible for structural components of cardiomyocyte plasma and organelle membranes as well as fuelling cardiac energy production, and are important players in the pathophysiological processes of various CVD [1, 16–18].

Lipidomics has emerged as an important comprehensive technology to quantify changing lipids in the human organism and analyze their contribution to various disease mechanisms [19–21]. A systematic analysis of circulating lipid species using a quantitative and reliable lipidomics technique (4D-LC/TIMS-IMS) offers a powerful tool to uncover metabolic shifts associated with SGLT2 inhibition, providing insight into the mechanisms underlying their cardiometabolic benefits. By enabling comprehensive profiling of lipid species, lipidomics can reveal alterations in lipid metabolism, energy homeostasis, and inflammatory pathways influenced by SGLT2 inhibitors [22, 23]. For example, changes in triglycerides, phospholipids, and sphingolipids may reflect shifts in substrate utilization and insulin sensitivity. Additionally, lipidomic analysis can identify novel biomarkers associated with cardiovascular and renal protection, shedding light on how SGLT2 inhibitors modulate systemic metabolism beyond glucose control. Integrating lipidomics into SGLT2 inhibitor research thus has the potential to deepen our understanding of their therapeutic effects and guide personalized treatment strategies.

## Methods

### EmDia study

The EmDia study is a randomized, double-blind, two-armed, placebo-controlled, parallel-group, investigator-initiated, phase IV study conducted by the University Medical Center of the Johannes Gutenberg University Mainz as the study sponsor. For information about study design, objectives, endpoints, as well as inclusion and exclusion criteria, please refer to Jünger et al.’s article on the design of the EmDia study [13]. For this analysis, plasma lipids at baseline, after one and twelve weeks of treatment with a daily oral dose of 10 mg Empagliflozin in addition to the concomitant medication were measured in 144 individuals with 4D-LC-TIMS/IMS lipidomics.

### Lipidomics measurement using 4D-LC-TIMS/IMS

The samples were acquired using a tandem Elute UPLC-TIMS-TOF pro instrument (Bruker Daltonics, Germany) in negative and positive modes. The instrumental parameters used for the data acquisition were as described in Lerner et al. [24]. Identification and annotation of the lipid features were based on characteristic fragment ions of each lipid feature from its MS2 spectra. Lipid classes such as ceramide (Cer), phosphatidylcholin (PC), phosphatidy-lethanolamin (PE), phosphatidylinositol (PI), and sphingomyelin (SM) were annotated in negative mode, while the Lipid classes such as cholesterol, triglyceride (TG), and diglyceride (DG) were annotated in positive mode. Lipid quantification was performed using a multi-point strategy based on external calibration standards [24]. Certain Lipid classes, such as PC, LPC, and SM were observed in both ionization modes. However, these lipid classes ionize better in positive polarity, and therefore, the quantified values of these commonly identified lipid species were from their positive adducts. Lipid quantification was performed using a multi-point strategy based on external calibration standards [24]. In total, 272 lipids were identified and quantified across the entire cohort.

Quality Control (QC) consisted of a phospholipid standard mixture run at the beginning, middle, and end of each batch (96 samples), with reproducibility (CV < 30%) manually checked using Bruker’s Compass data analysis software. Additionally, only targeted lipid features reproducible in NIST plasma SRM across the inter-day repetition study (Lerner et al.) from an untargeted DDA acquisition were included in this study. Furthermore, seven serial dilutions of a calibration mixture in each batch ensured the reproducibility of the selected lipid features [24].

### Lipidomics nomenclature

The lipid annotation follows the LIPID MAPS shorthand notation system, last updated in 2020 [25] as well as the IUPAC-IUBMB recommendations for biochemical nomenclature. Lipids were reported predominantly at the molecular species level, which includes identification of the individual fatty acyl/alkyl residues (e.g., PC 17:0_18:1). In cases where individual fatty acyl/alkyl residues could not be identified, the sum composition of total C-atoms and DBEs/DBs (double bond equivalents/double bonds) were reported instead (e.g., PC 34:5). When the stereospecific sn-positions of the residues were determined, these were indicated in the order sn1/sn2/sn3 using a slash (/) as separator, if the sn-position was unknown, an underscore (_) was used instead.

Generally, lipid names comprise the lipid class abbreviation (e.g., Cer, LPC, SM, TG) followed by the number of C-atoms, a colon (:), and the number of DBEs (replaced by DBs when experimentally confirmed). When additional oxygen atoms or other functional groups are present in the fatty acyl/alkyl residues, these are added after the DBE value and separated by a semicolon (;) (e.g., SM 34:0;3O). If present, ether linkages are denoted by the prefix O- (alkyl ether) preceding the number of C-atoms in the respective acyl/alkyl constituents within the nomenclature (e.g., PC O-22:2_18:2).

The general formula used is:

$$<\text{Lipid class}> <\#\text{C atoms}>:<\text{DBE}>[;<\#\text{O atoms}>]$$

### Statistical analysis

To select important features while considering group structures among them, the Least Absolute Shrinkage and Selection Operator (LASSO) was combined additively with the Group LASSO. The resulting penalty is known as Sparse Group LASSO, which balances group-level and individual feature selection to identify relevant groups while highlighting their most predictive members [26]. The tuning parameter α was set to 1/3, the default in the SGPR package, while λ was determined by tenfold-cross validation (CV) that optimizes the classification task between the placebo and Empagliflozin groups [27, 28]. Regularized regression models were used to investigate how the change in lipid signatures, identified regarding the influence of one and twelve weeks of Empagliflozin treatment respectively (based on the selection by the sparse group LASSO regularized models), is related to the change in clinical characteristics of the participants as well as how the change in the selected lipid profiles is related to the classification of the samples in selected clinical subgroups (left ventricular ejection fraction (LVEF) (≥ 55% or < 55%), NT-proBNP (< 125 pg/ml or ≥ 125 pg/ml), left ventricular hypertrophy (LVH), eGFR (< 60 ml/min/1.73 m^2^ or ≥ 60 ml/min/1.73 m^2^), obesity (BMI ≥ 30) [30], HbA1c (< 6.5% or 6.5–6.9%), uric acid (< 6 mg/dL or ≥ 6 mg/dL for females, < 7 mg/dL or ≥ 7 mg/dL for males)).

All data pre-processing and analysis were conducted using the statistical software package R, version 4.2.1 (R Foundation for Statistical Computing, Vienna, Austria; http://www.r-project.org).

## Results

Most clinical parameters at baseline of the Empagliflozin and placebo groups (e.g., smoking status, percentage of females, age) are comparable (Table 1). Nevertheless, to enable non-biased results as best as possible, following analyses were adjusted for sex, age as well as E/E′ at baseline. After one week of treatment weight, DBP, BMI, BSA, hematocrit, erythrocytes, hemoglobin, and several renal parameters were already affected by the intake of Empagliflozin (Supplementary Table 1) [7]. Treatment with Empagliflozin for 12 weeks improved several clinical parameters, namely E/E′, weight, BMI, BSA, HbA1c, hematological traits, and other traits related to kidney function (Supplementary Table 2) [7].

**Table 1:** Sample characteristics at baseline of the EmDia study. Baseline characteristics table of clinical parameters, including age, sex, cardiovascular risk factors, echocardiography and clinical profile

Clinical parameter	Empagliflozin (n = 72)	Placebo (n = 72)
Age [years], mean ± SD	69.3 ± 7.4	68.5 ± 8.0
Female sex, [%] (n)	15.5 (11)	12.7 (9)
*Cardiovascular risk factors*		
Arterial hypertension, [%] (n)	84.5 (60)	94.4 (67)
Dyslipidemia, [%] (n)	84.5 (60)	90.1 (64)
Family history of MI or stroke, [%] (n)	28.2 (20)	32.4 (23)
Obesity, [%] (n)	64.8 (46)	57.7 (41)
Smoking (current), [%] (n)	12.7 (9)	15.5 (11)
*Echocardiography*		
LVEF [%, mean ± SD]	59.2 ± 5.5	57.9 ± 5.7
E/E′ ratio [median, IQR]	9.90 (8.40/11.90)	9.06 (8.14/10.30)
LVEDV [mL, mean ± SD]	112.7 ± 28.9	115.8 ± 28.3
*Clinical profile*		
Atrial fibrillation, [%] (n)	23.2 (16)	29.9 (20)
Chronic kidney disease, [%] (n)	13.0 (9)	20.0 (14)
COPD, [%] (n)	7.1 (5)	5.6 (4)
Congestive HF, [%] (n)	17.5 (11)	28.8 (19)
Coronary artery disease, [%] (n)	35.9 (23)	44.1 (30)
History of MI, [%] (n)	23.2 (16)	37.1 (26)
History of TIA/stroke, [%] (n)	7.1 (5)	9.6 (7)
History of VTE, [%] (n)	7.1 (5)	10.0 (7)
Peripheral artery disease, [%] (n)	10.1 (7)	11.9 (8)

*LVEF* left ventricular ejection fraction;* E/E’* ratio between early mitral inflow velocity and mitral annular early diastolic velocity;* LVEDV* left ventricular end‐diastolic volume,* COPD* chronic obstructive pulmonary disease;* MI* myocardial infarction;* TIA* transient ischemic attack;* VTE* venous thromboembolism;* SD* standard deviation;* IQR* interquartile range

### Lipid signatures after one and twelve weeks of treatment with Empagliflozin

Using sparse-group LASSO regularized regression models, Empagliflozin-associated lipids were selected to generate signatures for treatment with Empagliflozin after one and twelve weeks, respectively (using the difference in value, or delta, between baseline and one as well as the delta between baseline and twelve weeks).

**Fig. 1:**
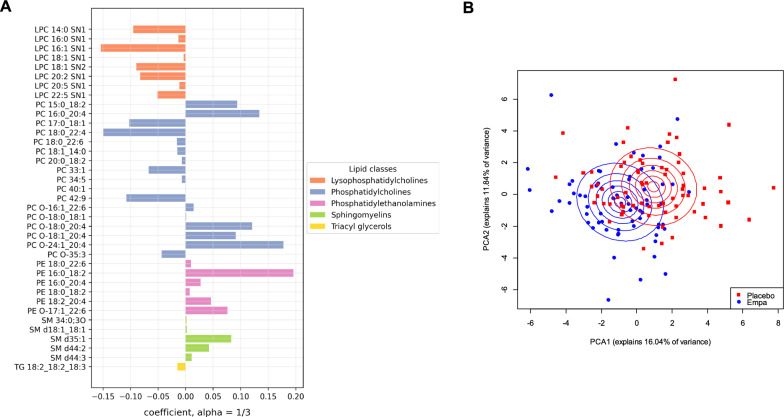
Sparse group LASSO regularized regression model of the lipid-signature after one week of Empagliflozin treatment. **A** Barplot showing the 37 individual lipids of the lipid classes LPC, PC, PE, SM and TG that were selected by sparse group LASSO regularized regression model as being significantly associated with Empagliflozin treatment after one week. **B** PCA plot for the selected 37 lipids by the sparse group LASSO regularized model, showing an initial separation of treatment (Empagliflozin vs placebo) clusters beginning to emerge after one week

**Fig. 2:**
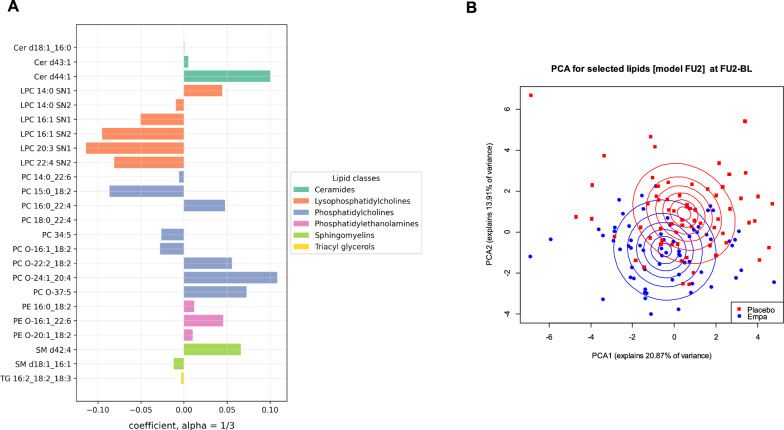
Sparse group LASSO regularized regression model of the lipid-signature after twelve weeks of Empagliflozin treatment. **A** Barplot showing the 24 individual lipids of the lipid classes LPC, PC, PE, SM, TG and Cer that were selected by sparse group LASSO regularized regression model as being significantly associated with Empagliflozin treatment after twelve weeks. **B** PCA plot for the selected 24 lipids by the sparse group LASSO regularized model, showing a distinct separation of treatment clusters (Empagliflozin vs. placebo) after twelve weeks

The lipid signature after treatment for one week was comprised of 37 lipids of the five lipid groups LPC, PC, PE, SM, and TG (Fig. 1A). Empagliflozin reduces all selected LPCs and TGs of the signature, although with differing amplitudes, and increases all the selected PEs and SMs. PCs show various responses to treatment. Except for PC O-35:3, all ethylated PCs increase upon Empagliflozin treatment. Similarly, while PC 15:0_18:2, PC 16:0_20:4, and PC 42:9 increase, all other PCs decrease after one week of Empagliflozin treatment. After twelve weeks of treatment, the Empagliflozin signature consisted of 24 lipids from six lipid groups, five of which were also present in the one-week signature, with the addition of Cers **(**Fig. 2A**)**. Empagliflozin increased all Cers and PEs, and decreased all TGs, while it resulted in mixed effects in LPCs, PCs, and SMs. Specifically, with the exception of LPC 14:0 SN1, all other LPCs (LPC 14:0 SN2, LPC 16:1 SN1, LPC 16:1 SN2, LPC 20:3 SN1, LPC 22:4 SN2) decreased. Certain PCs like PC 14:0_22:6, PC 15:0_18:2, and PC 34:5 were decreased, while PC 16:0_22:4, PC O-22:2_18:2, PC O-24:1_20:4, and PC O-37:5 increased. Treatment also caused an increase in SM d42:4 and a decrease in SM d18:1_16:1.

Interestingly, only five lipids are included in both signatures affected by Empagliflozin treatment. Among them, the changes of LPC 16:1 SN1, PC 34:5, and PE 16:0_18:2 were consistent in both signatures, while LPC 14:0 SN1 and PC 15:0_18:2 exhibit discordant effects between the two timepoints.

Two sets of PCAs based on first the 37 lipid species and then the 24 lipids selected after one and twelve weeks of treatment by the sparse group LASSO, respectively, were performed to confirm the validity of our models. As can be seen in both sets of PCAs after one and twelve weeks, no differences could be observed between the placebo and Empagliflozin treated individuals at baseline (Supplementary Fig. 1A and B), while an initial separation of treatment clusters is beginning to emerge after one week of treatment (Fig. 1B), and a distinct separation of treatment clusters after twelve weeks **(**Fig. 2B**)** can be seen (for PCA parameters see Supplementary Tables 3–6).

An evaluation of the diagnostic performance of individual lipids was carried out as well, using Receiver Operating Characteristic (ROC) analysis based on the sparse group LASSO regularized regression models. Using a naïve cutoff of 0.5, the ROC analysis after one week of treatment provided us with a sensitivity of 0.797, a specificity of 0.899, and an AUC of 0.923 (Supplementary Fig. 2A). ROC analysis after twelve weeks of treatment (naïve cutoff of 0.5) showed a sensitivity of 0.723, a specificity of 0.794, and an AUC of 0.864 (Supplementary Fig. 2B). These results suggest that our models provide excellent discrimination after one week of treatment and a good discrimination after 12 weeks (although slightly less so compared to the one week timepoint). The sensitivity and specificity values further support the robustness of our sparse group LASSO models in identifying true positives and avoiding false positives.

Additionally, we performed multiple linear regression models after Empagliflozin treatment for one and twelve weeks, respectively, based on the delta of lipids and adjusted for sex, age, and E/E′ at baseline (same covariates as in the sparse group LASSO models). We performed two types of* p*-value correction, with the lipids LPC 16:1 SN1 (0.061 [0.016; 0.241],* p*-value: 0.018) and LPC 14:0 SN1 (0.112 [0.034; 0.369], *p*-value: 0.044) remaining significant after FDR correction and only LPC 16:1 SN1 (*p*-value: 0.018) being left as significant after Bonferroni correction after treatment with Empagliflozin for one week (Supplementary Table 7).

Similarly, after FDR correction, LPC 16:1 SN1 (0.083 [0.021; 0.324], *p*-value: 0.048) as well as LPC 16:1 SN2 (0.104 [0.031; 0.343], *p*-value: 0.048) remained significant, while after Bonferroni correction no lipid species were left as significant after treatment for twelve weeks (Supplementary Table 8).

Both LPC 16:1 SN1 (the only lipid remaining as showing a significant association with Empagliflozin after one week), as well as the lipids LPC 16:1 SN1 and LPC 16:1 SN2, were selected by our sparse group LASSO regularized regression models and show the same direction of association (a negative association) as well.

### Association between selected clinical traits and Empagliflozin-associated lipids after one and twelve weeks of treatment with Empagliflozin

Several clinical traits were closely examined due to their notable changes between baseline and Empagliflozin treatment for one and twelve weeks, respectively (Supplementary Tables 1 and 2) [7].

After one week of Empagliflozin treatment, hematocrit, estimated glomerular filtration rate (eGFR), BMI, diastolic blood pressure (DBP), uric acid, erythrocytes, phosphate, albumin, total proteins, weight, body surface area (BSA), as well as creatinine were significantly different in Empagliflozin-treated compared to placebo-treated participants (Supplementary Table 1).

**Fig. 3:**
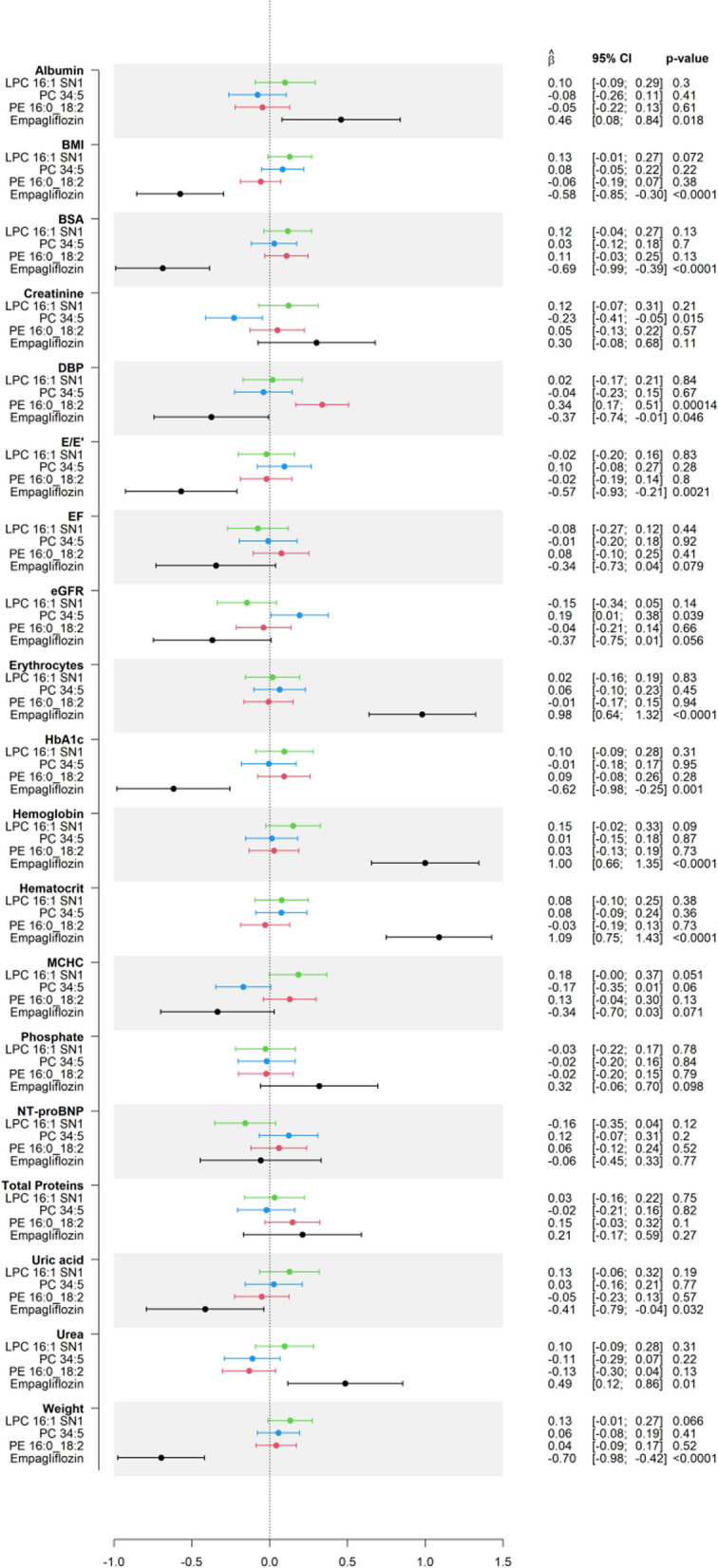
Forest-plot of the association of individual lipids with selected subgroups after Empagliflozin treatment for twelve weeks. Using linear regression with Bonferroni-correction (threshold = 0.000183), a forest plot was generated to show the association of individual lipids with selected clinical subgroups after Empagliflozin treatment for one week (adjusted by sex, age and E/E′ at baseline)

Analysis of the linear model with Empagliflozin and the three lipids consistently changed by Empagliflozin across the two time points (LPC 16:1 SN1, PC 34:5, PE 16:0_18:2) allows to investigate to what extent the Empagliflozin-mediated lipids explain differences in clinical traits between treatment groups at one and twelve weeks of treatment. At one week, BMI and weight are decreased by Empagliflozin and increased by PC 34:5 (which is consistently decreased by Empagliflozin at both time points). This data shows a consistent and synergistic effect of Empagliflozin and its regulated lipid PC 34:5 in regulating body weight (Supplementary Fig. 3). At twelve weeks, DBP is significantly decreased by Empagliflozin and increased by PE 16:0_18:2 (Fig. 3), demonstrating a negative feedback regulation between an Empagliflozin-mediated clinical outcome and the effect of an Empagliflozin-regulated lipid species. Associations between lipid species and clinical traits in a larger cohort is needed to disentangle this phenomenon.

To gain a better understanding of the effects of the lipids consistently regulated by Empagliflozin within the Empagliflozin and placebo treatment groups, we have performed analyses of the association between these lipids and clinical traits at twelve weeks (Table 2 and Supplementary Table 9). In the Empagliflozin group, E/E′ is positively associated with PC 34:5, which is consistently decreased by Empagliflozin at the two time points, suggesting that this lipid might be a contributor to the Empagliflozin-mediated decrease in E/E′ (Table 2). Similarly, LPC 16:1 SN1, another lipid decreased with Empagliflozin, is positively associated with BMI, indicating that this lipid might synergistically work with Empagliflozin to decrease body weight.

**Table 2:** Association between clinical traits and lipids within the Empagliflozin treatment group after 12 weeks of treatment

Clinical trait	Lipid	Coefficient for lipid in sparse group LASSO model, α = 1/3	Estimate [95% CI]	*p*-value	Group
*Cardiac function and biomarkers*					
E/E′	PC 34:5	− 0.026	0.722 [0.024; 1.219]	0.043	Empagliflozin
*Renal function*					
Creatinine	PE 16:0_18:2	0.012	0.079 [0.01; 0.149]	0.026	Empagliflozin
eGFR	PE 16:0_18:2	0.012	− 4.886 [− 9.724; − 0.048]	0.048	Empagliflozin
*Physiological and circulatory health*					
MCHC	PE 16:0_18:2	0.012	0.531 [0.069; 0.993]	0.025	Empagliflozin
BSA	PE 16:0_18:2	0.012	0.017 [0.004; 0.029]	0.0087	Empagliflozin
DBP	PE 16:0_18:2	0.012	6.801 [2–837; 10.764]	0.0011	Empagliflozin
BMI	LPC 16:1 SN1	− 0.051	0.581 [0.01; 1.151]	0.046	Empagliflozin
Total proteins	PE 16:0_18:2	0.012	2.895 [0.115; 5.676]	0.042	Empagliflozin

Lipids with consistent Empagliflozin-driven change in abundance among timepoints were used as covariates in a model in which changes in clinical traits between baseline and twelve weeks within individuals in the Empagliflozin group were used as outcome

Notably, both in the placebo- and Empagliflozin-treated groups, DBP is positively regulated by PE 16:0_18:2 (Table 2 and Supplementary Table 9). Empagliflozin increases this lipid, and the drug decreases DBP at twelve weeks when including the three Empagliflozin-modulated lipids (Fig. 3); therefore, we can conclude that the PE 16:0_18:2 affects DBP independently of Empagliflozin.

Although the cohort does not have enough statistical power to associate all lipids with clinical traits, we have performed this analysis at each time point and discovered that after one week, following Bonferroni correction, only erythrocytes were found to be significantly linked with lipids, specifically with the increase of PE 18:0_22:6 (Supplementary Table 10).

A detailed investigation was also conducted on various clinical traits that exhibited significant changes between baseline and a twelve-week treatment. Among these traits, BMI, weight, and BSA were found to be significantly associated with lipids (Supplementary Table 11). Both BMI as well as weight showed a positive connection to LPC 16:1 SN1, while both weight and BSA also exhibited a positive association with LPC 14:0 SN1 (Table 3).

**Table 3:** Table of associations of individual lipids with clinical traits after Empagliflozin treatment for one and twelve weeks

Clinical trait	Lipid	Beta-estimate	*p*-value	Timepoint
Erythrocytes	PE 18:0_22:6	0.0650	0.00016	After 1 week
BMI	LPC 16:1 SN1	0.233	0.00013	After 12 weeks
Weight	LPC 14:0 SN1	0.730	0.000016	After 12 weeks
	LPC 16:1 SN1	0.716	0.000020	After 12 weeks
BSA	LPC 14:0 SN1	0.00752	0.000065	After 12 weeks

Table of associations between individual lipids and selected clinical traits that showed significant changes after one and twelve weeks respectively. Linear regression was used to find these associations, and Bonferroni correction (threshold = 0.000183) was used (complete analysis of all traits in Supplementary Tables 9 and 10)

### Association between the change in Empagliflozin-associated lipids and selected clinical subgroups after one and twelve weeks of treatment

Based on the previous clinical analysis of the EmDia study [7], specific clinical traits were selected to subgroup patients and identify Empagliflozin-associated lipids specific for each clinical subgroup. This analysis was performed both for treatment with Empagliflozin after one and twelve weeks, respectively. Clinical traits that were selected included LVEF (≥ 55% or < 55%), NT-proBNP (< 125 pg/mL or ≥ 125 pg/mL), LVH, eGFR (< 60 mL/min/1.73 m^2^ or ≥ 60 mL/min/1.73 m^2^), obesity (BMI ≥ 30), HbA1c (< 6.5% or 6.5–6.9%), and uric acid (< 6 mg/dL or ≥ 6 mg/dL for females, < 7 mg/dL or ≥ 7 mg/dL for males).

Analysis of the Empagliflozin-associated lipids after one week of treatment revealed distinct lipid profiles associated with various clinical subgroups (Supplementary Fig. 4). For individuals with preserved LVEF (LVEF ≥ 55%), LPC 14:0 SN1 and LPC 16:1 SN1 were negatively correlated. Among patients with NT-proBNP levels ≥ 125 pg/mL, PC 17:0_18:1 showed a negative association. In cases of patients suffering from LVH, LPC 14:0 SN1, LPC 16:0 SN1, and LPC 16:1 SN1 were negatively associated. Individuals with an eGFR of ≥ 60 mL/min/1.73 m^2^ exhibited negative associations of LPC 14:0 SN1 and LPC 16:1 SN1, in line with the lipid profile. Both patients with obesity, as well as HbA1c levels between 6.5% and 6.9% were linked to a negative association of LPC 16:1 SN1. Uric acid levels below the defined threshold (< 6 mg/dL for females and < 7 mg/dL for males) were associated with negative correlations of LPC 14:0 SN1, LPC 15:0 SN1, and LPC 16:1 SN1. The effects on all the lipids tested align with the overall lipid signature one week after Empagliflozin treatment.

Notably, two lipids (LPC 14:0 SN1 and LPC 16:1 SN1) emerged as significant across multiple clinical subgroups including in patients with preserved LVEF, LVH, an eGFR of ≥ 60 mL/min/1.73 m^2^, obesity (only LPC 16:1 SN1), HbA1c levels between 6.5 and 6.9%, and uric acid levels below the threshold.

Lipid profiles associated with various clinical traits across different subgroups could also be generated for Empagliflozin-associated lipids after twelve weeks of treatment (Supplementary Fig. 5). LPC 16:1 SN2 was negatively associated in the clinical subgroup with preserved LVEF (≥ 55%), with LVH and HbA1c levels between 6.5 and 6.9%. Patients with an eGFR of ≥ 60 mL/min/1.73 m^2^ also showed negative associations of LPC 14:0 SN2 as well as LPC 16:1 SN2. An analysis of patients with differing uric acid levels did not display any significant associations. Interestingly, all significant associations with lipids that could be observed at this time point were linked to only two lipids, namely LPC 14:0 SN2 and LPC 16:1 SN2.

## Discussion

In this work, we explored the contribution of Empagliflozin on circulating plasma lipid levels and provided insights into the role of these lipids in human health.

The advantage of our approach to clinical lipidomics analysis lies within the 4D-LC-TIMS/IMS method [24], which makes it possible to analyze lipidomics samples rapidly in a high-throughput capacity and granularity [24]. In utilizing a 4-dimensional approach, information about retention time, collision cross-section, mass-to-charge-ratio (*m/z*), and MS/MS spectra is collected for each measured lipid to enable a very in-depth and detailed analysis of the measured lipidomics samples. Further, with the combination of improved sensitivity, reproducibility, highly reliable data quality, and robust annotation, the false discovery rate in lipid identification is reduced [24].

Analysis of the lipid signatures after one and twelve weeks of treatment with Empagliflozin has shown a dynamic change in lipid levels following treatment. While there was a greater number of significant lipids associated with treatment after one week, diversity increased after twelve weeks. Comparing the individual lipids of the Empagliflozin signatures also shows that three of the five overlapping lipids between both time points, LPC 16:1 SN1, PC 34:5, and PE 16:0_18:2, change in the same direction with treatment within the tested time points. On the other hand, LPC 14:0 SN1 showed a negative relationship with treatment after one week, which changed to a positive association after treatment for twelve weeks. In contrast, PC 15:0_18:2 increased after one week of Empagliflozin treatment, while it decreased after twelve weeks of treatment. The reason behind both observations might be that these lipids are produced by different tissues or by physiological processes that are dynamically changing over time after Empagliflozin treatment. Due to the paucity of lipidomics analyses with the same depth as the 4D-LC-TIMS/IMS method, the association of all the lipids described in this study with physiological or pathological processes in the human organism is unfortunately not yet described in detail. Therefore, the reason behind the dynamic changes of lipids across treatment time points can only be speculated. Still, the observation of only a small subset of lipids overlapping between the signatures of the two timepoints highlights the dynamic nature of lipid metabolism in response to Empagliflozin treatment and might reflect tissue-specific lipid turnover, adaptive metabolic responses, or feedback mechanisms that evolve with longitudinal therapy.

Comparison of individual lipids with the current state of knowledge proved to be difficult. On one side, there’s not much currently known about the association of individual lipid species with clinical traits or molecular mechanisms, and on the other side, not all lipid species belonging to the same lipid class were regulated in a coordinated manner.

When considering the lipid classes that comprise the signatures, it is surprising to see that Empagliflozin treatment seems to be associated with an increase in ceramides. Ceramides are generally significantly connected to CVD through contributions to various pathological processes, including inflammation in atherosclerosis, metabolic abnormalities (e.g., insulin resistance), and diabetic cardiomyopathy. In particular, Cer d18:1_16:0 showed a correlation with liver and kidney dysfunction while promoting inflammatory activation during cardiac dysfunction in patients with worsening of HF [29]. Though several authors suggest that lowering ceramide levels is a valid strategy to battle the onset and progression of CVD [30–34], Empagliflozin does not seem to support improvement of the HF syndrome through this mechanism.

It has been shown that LPCs impact cell functions via G-coupled protein receptors (GPCRs), influencing vascular reactivity and playing roles in both pro-inflammatory and anti-inflammatory processes, affecting endothelial function and immune cell behaviour, but they also possess anti-inflammatory properties, such as inhibition of platelet aggregation and enhanced antioxidant capacity. Decreased plasma LPC levels have been associated with unfavorable outcomes in diseases like rheumatoid arthritis, diabetes, and cancer, and are linked to the development of atherosclerotic plaques, myocardial infarction, and insulin resistance, contributing to CVD progression [35–37]. In our study, Empagliflozin mostly decreased LPCs. The negative outcomes of LPC decrease suggest that this Empagliflozin-mediated change in the lipidome is likely not the mechanism through which Empagliflozin exerts its positive effects. However, as the optimal plasma LPC level remains uncertain, it is rather difficult to ascertain the impact of LPC modulation by Empagliflozin [35–37].

Similarly, it’s also difficult to explain the connection between Empagliflozin treatment and the various different associations with PCs and the increase in PEs. Both abnormally high and low ratios of PCs and PEs have been connected to disease progression in multiple organs, including affecting insulin sensitivity and glucose tolerance. However, the presence of both PCs and PEs has also been implicated in the metabolism of very low-density lipoproteins (VLDLs) [38, 39].

Not much is known about the association between SMs specifically with HF. According to Hammad et al., a higher level of sphingomyelins in plasma might act as a risk factor for coronary heart disease [40]. However, the fatty acid chain length and composition of the sphingolipid appear to influence the relative contribution. Additionally, Kovilakath et al. mention that while ceramides (which are sphingolipids with a hydrogen molecule as the head group) are associated with an increased HF risk, SMs show inverse correlations [41]. This would suggest that Empagliflozin might ameliorate HF through SMs, as our results show that Empagliflozin treatment promotes an increase in SMs.

Viewing of the association between selected clinical traits and Empagliflozin-associated lipids after one and twelve weeks of treatment provided hints on the role of some lipids as biomarkers of clinical traits. After treatment for one week, only the variation in erythrocyte numbers showed a significant connection to lipids, PE 18:0_22:6 in this case. After treatment for twelve weeks, three clinical traits were associated with lipids: body weight, BMI as well as BSA. However, as both BMI and BSA are calculated using the variable body weight, it is not surprising that the two significant lipids for body weight, LPC 14:0 SN1 and LPC 16:1 SN1, are also associated with BSA and BMI, respectively. It should also be noted that the EmDia study, with 144 participants, might not provide sufficient statistical power to detect other significant associations of clinical traits with lipids. Future analyses of the plasma 4D-LC-TIMS/IMS lipidomes in larger cohorts will generate significant connections between clinical traits and lipids, and this new knowledge can retrospectively provide more insights into the results of this study.

Analyzing the association between the change in Empagliflozin-associated lipids and selected clinical subgroups offered fascinating insights. Of the 37 lipids of the lipid signature after one week of treatment, only four of them are significantly associated with clinical subgroups of relevance for HF. Notably, among these, two lipids appear to be significantly associated with several relevant clinical subgroups. Both LPC 14:0 SN1 and LPC 16:1 SN1 are negatively associated with the subgroups of patients that were diagnosed with either preserved ejection fraction, LVH, and obesity, or showed an eGFR ≥ 60 mL/min/1.73 m^2^, HbA1c between 6.5 and 6.9% or a uric acid level below the defined threshold.

Similar results could be observed in the analysis of subgroups after twelve weeks of treatment. Of the 24 lipids of the lipid signature, only two lipids were significantly associated with subgroups of patients, namely preserved ejection fraction, LVH, an HbA1c between 6.5 and 6.9%, or an eGFR ≥ 60 mL/min/1.73 m^2^.

Again, LPCs seem to be of especially interest associated with Empagliflozin treatment, with LPC 14:0 SN2 and LPC 16:1 SN2 being negatively associated with the mentioned subgroups after twelve weeks. This repeated association of those two lipids with multiple clinical subgroups may point to a central role in the metabolic response to Empagliflozin, warranting further mechanistic investigation.

Overall, the performance of the lipid signatures in differentiating Empagliflozin from placebo proved to be robust and had a strong discriminatory power, as evidenced by the results of the PCAs and ROC analyses. The PCAs showed no baseline separation but revealed clear treatment group separation after both one and twelve weeks, respectively. ROC analyses also demonstrated excellent diagnostic performance with high sensitivity, specificity, and AUC after both treatment timepoints.

These results offer interesting insights, as we have identified Empagliflozin-driven lipids that serve as biomarkers of clinical subgroups. As the participants of the EmDia study have a relatively homogenous clinical profile, further investigations should be conducted to identify all lipids relevant to clinical subgroups. Additionally, as already mentioned, future in-depth analysis of lipidomes in larger cohorts, longitudinal studies, and functional validation of possible lipid biomarkers would be of interest.

Interestingly, the effect of some lipids on clinical outcomes was discordant with what we would expect if they were exclusive mediators of Empagliflozin action, and it is possible that, on their own, they have an effect on several clinical outcomes that bypass Empagliflozin-mediated regulation. Larger, treatment-corrected cohorts will be required to clarify the complex relationships between certain lipids and clinical traits independently of Empagliflozin.

Generally, mass spectrometry-based lipidomics remains primarily a research tool rather than a routine clinical assay, but it holds significant potential for clinical translation in the future. Lipidomic profiling could reveal metabolic heterogeneity within populations of T2DM and HFpEF, helping to identify patient subgroups that may benefit more from SGLT2 inhibitor therapy [42]*.* Supporting this, large-scale clinical trials such as the EMPEROR-Preserved trial already demonstrated that the benefit of Empagliflozin on cardiovascular outcomes varies according to LVEF, highlighting clinical heterogeneity in HFpEF patients and the need for more precise patient stratification strategies [43, 44]

Overall, integration of lipidomics with clinical phenotyping and other omics data offers a promising path toward identifying molecular HFpEF and T2DM subtypes with differential SGLT2i responses, thus guiding precision medicine approaches.

An example of this translational potential can be found in the mCVDRisk score, which combines lipidomic and genetic profiling to better stratify cardiovascular risk, especially in individuals classified as being at “intermediate” risk by traditional methods [45]*.* Although still under validation and not yet standard clinical practice, this score represents a promising step towards the clinical applicability of lipidomics. Nevertheless, a deeper understanding of individual lipid species’ biological roles, along with larger prospective studies and the standardization of lipidomic assays, remains critical before broader clinical implementation.

## Conclusion

We successfully identified distinct lipid signatures associated with Empagliflozin treatment at one and twelve weeks in the EmDia study. Specifically, 37 lipids were significantly altered after one week of treatment, while a signature of 24 lipids was observed after twelve weeks, with five lipids overlapping between these time points. This temporal variation underscores the dynamic nature of lipidomic responses to Empagliflozin therapy.

Our study reveals that Empagliflozin induces selective modulation of key lipid classes—ceramides, LPCs, PCs, PEs, and SMs—with distinct temporal patterns and clinical associations in heart failure patients. While ceramide increases challenge conventional paradigms, the modulation of LPCs and SMs points to novel pathways potentially underlying Empagliflozin’s cardiometabolic benefits.

While establishing direct causal relationships between individual lipid species and clinical outcomes remains challenging, our data indicate that analyzing lipid classes as functional units provides valuable insights into the lipidomic mechanisms of Empagliflozin. This approach may overcome the heterogeneity observed at the single lipid species level and better capture biologically relevant patterns.

Overall, our study underscores both the complexity and the promising potential of advanced lipidomics to deepen our understanding of the mechanisms underlying drugs such as Empagliflozin, as well as to identify novel biomarkers. To fully elucidate the precise molecular pathways involved, further mechanistic investigations are essential. Additionally, exploring these lipid species as potential biomarkers or therapeutic targets could significantly advance heart failure management. In particular, the application of advanced technologies such as 4D-LC-TIMS/IMS lipidomics in larger and more diverse cohorts will be critical to clarifying the roles of these lipids in cardiometabolic health and to further unraveling the pathways through which Empagliflozin exerts its clinical benefits.

These findings highlight the promise of lipidomics in understanding drug effects and patient heterogeneity. Alongside studies on how lipids affect the body and their potential as markers for disease, new clinical tools (e.g., the mCVDRisk score) show the potential for lipidomics to inform personalized clinical care in the future.

## Supplementary Information

Supplementary file 1.

Supplementary file 2.

Supplementary file 3.

## Data Availability

This project constitutes a major scientific effort with high methodological standards and detailed guidelines for analysis and publication. Data are not made available for the scientific community outside the established and controlled workflows and algorithms. To meet the general idea of verification and reproducibility of scientific findings, we offer access to data at the local database in accordance with the ethics vote on request (contact: Univ.-Prof. Dr. Philipp Wild (principal investigator of the EmDia Study), philipp.wild@unimedizin-mainz.de).

## Abbreviations

BMI: Body mass index
BSA: Body surface area
Cer: Ceramide
CVD: Cardiovascular disease
DB: Double bond
DBE: Double bond equivalents
DBP: Diastolic blood pressure
E/E′: Ratio of early diastolic mitral inflow velocity to early diastolic mitral annulus velocity
eGFR: Estimated glomerular filtration rate
GPCR: G-coupled protein receptor
HbA1c: Glycosylated hemoglobin
HF: Heart failure
HFpEF: HF with preserved ejection fraction
LASSO: Least Absolute Shrinkage and Selection Operator
LPC: Lysophosphatidylcholin
LVEF: Left ventricular ejection fraction
LVH: Left ventricular hypertrophy
*m/z*: Mass-to-charge-ratio
MCHC: Mean cell hemoglobin concentration
PC: Phosphatidylcholine
PE: Phosphatidylethanolamine
PI: Phosphatidylinositol
RBC: Red blood cell
ROC: Receiver operating characteristic
SGLT2: Sodium-glucose cotransporter 2
SGLT2i: SGLT2 inhibitor
SM: Sphingomyelin
SS loadings: Sum of squared loadings
TG: Triacylglycerol
VLDL: Very low-density lipoprotein
