# Supplementary material for: Effect of Empagliflozin on the plasma lipidome in patients with type 2 diabetes mellitus: results from the EmDia clinical trial
Source: Cardiovasc Diabetol. 2025 Sep 8;24:359. doi: 10.1186/s12933-025-02916-0 (PMC12418620; doi:10.1186/s12933-025-02916-0)

Supplementary Figure 1

A

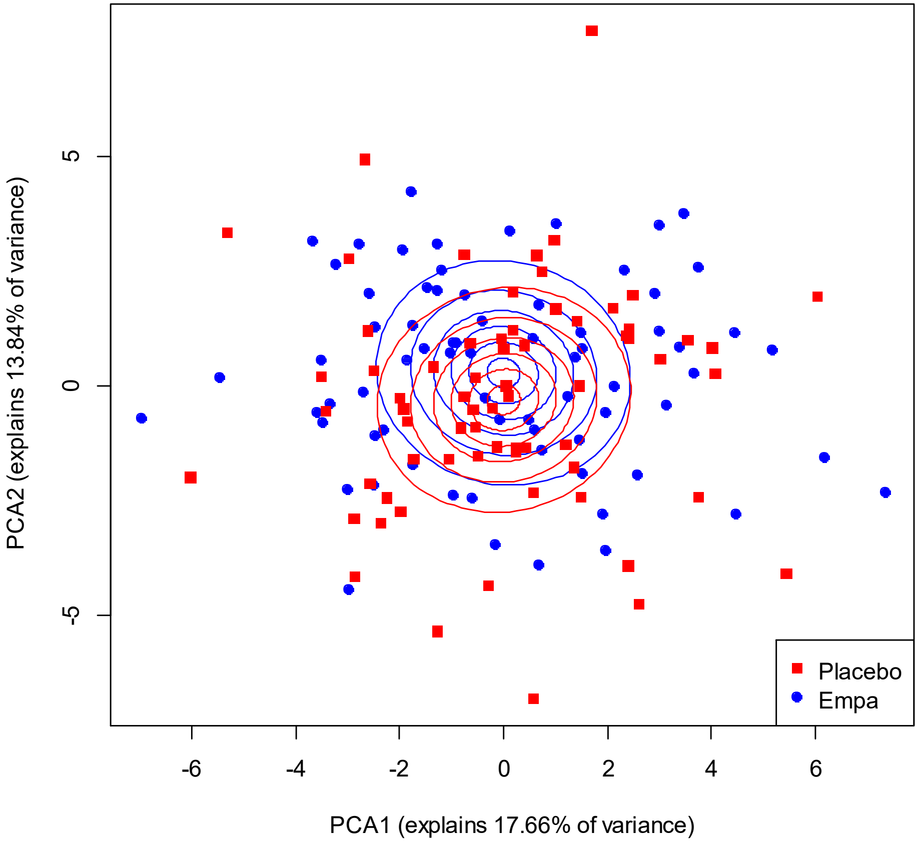

B

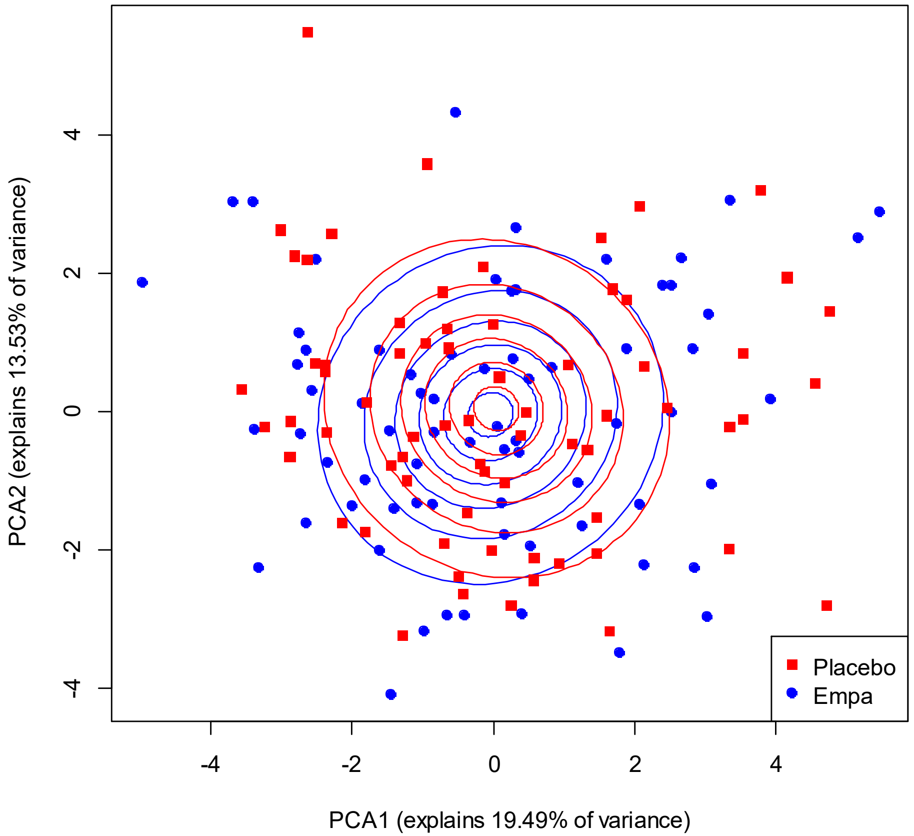

Supplementary Figure 2

A

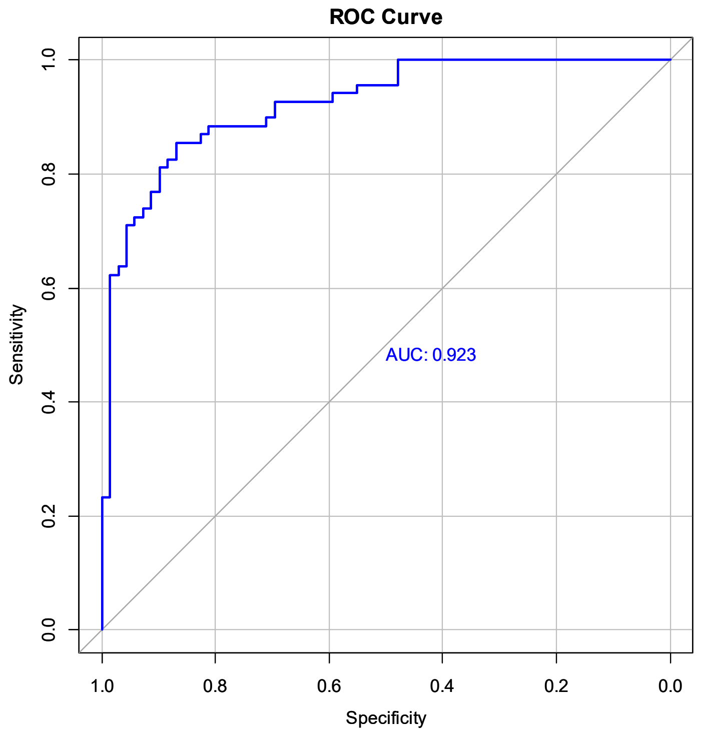

B

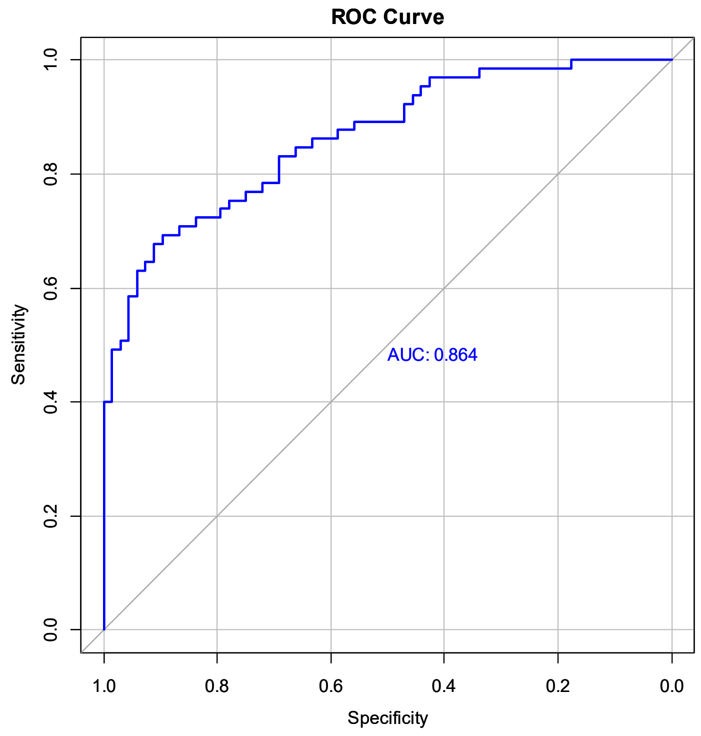

Supplementary Figure 3

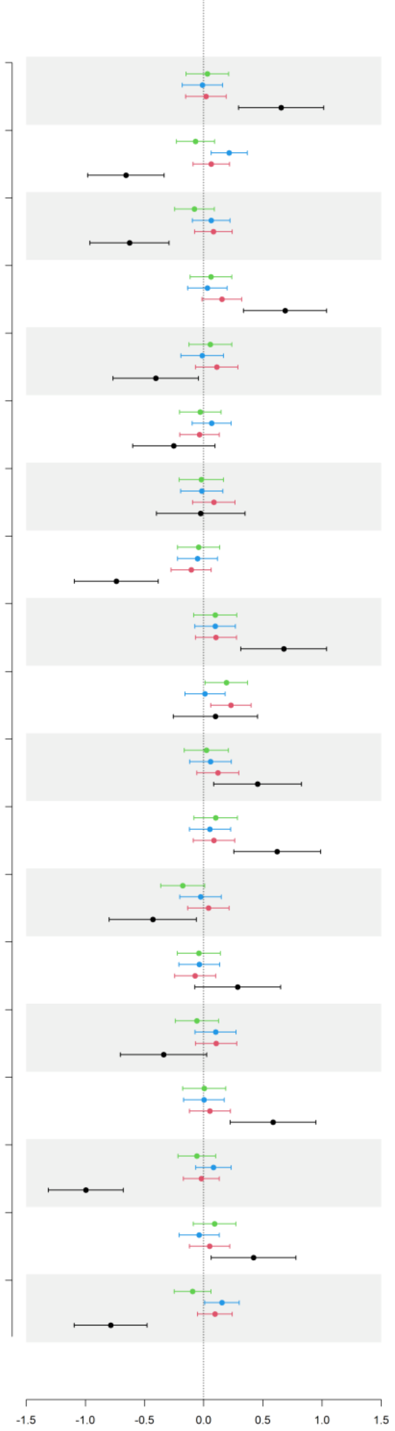

		$\hat{\beta}$	95% CI	p-value
Albumin	LPC 16:1 SN1	0.03	[-0.15; 0.21]	0.73
	PC 34:5	-0.01	[-0.18; 0.16]	0.91
	PE 16:0_18:2	0.02	[-0.15; 0.19]	0.82
	Empagliflozin	0.66	[0.30; 1.01]	0.00045
BMI	LPC 16:1 SN1	-0.07	[-0.23; 0.09]	0.4
	PC 34:5	0.22	[0.06; 0.37]	0.006
	PE 16:0_18:2	0.06	[-0.09; 0.22]	0.41
	Empagliflozin	-0.66	[-0.98; -0.34]	<0.0001
BSA	LPC 16:1 SN1	-0.08	[-0.25; 0.09]	0.36
	PC 34:5	0.06	[-0.10; 0.22]	0.43
	PE 16:0_18:2	0.08	[-0.08; 0.24]	0.31
	Empagliflozin	-0.63	[-0.96; -0.29]	0.00031
Creatinine	LPC 16:1 SN1	0.06	[-0.12; 0.24]	0.49
	PC 34:5	0.03	[-0.14; 0.20]	0.71
	PE 16:0_18:2	0.15	[-0.01; 0.32]	0.073
	Empagliflozin	0.69	[0.34; 1.04]	0.00017
DBP	LPC 16:1 SN1	0.06	[-0.13; 0.24]	0.54
	PC 34:5	-0.01	[-0.19; 0.17]	0.89
	PE 16:0_18:2	0.11	[-0.07; 0.29]	0.23
	Empagliflozin	-0.41	[-0.77; -0.04]	0.028
E/E'	LPC 16:1 SN1	-0.03	[-0.20; 0.15]	0.75
	PC 34:5	0.07	[-0.10; 0.23]	0.42
	PE 16:0_18:2	-0.04	[-0.20; 0.13]	0.68
	Empagliflozin	-0.25	[-0.60; 0.10]	0.15
EF	LPC 16:1 SN1	-0.02	[-0.21; 0.17]	0.83
	PC 34:5	-0.02	[-0.20; 0.16]	0.85
	PE 16:0_18:2	0.09	[-0.09; 0.27]	0.34
	Empagliflozin	-0.03	[-0.40; 0.35]	0.9
eGFR	LPC 16:1 SN1	-0.04	[-0.22; 0.14]	0.64
	PC 34:5	-0.05	[-0.22; 0.12]	0.55
	PE 16:0_18:2	-0.11	[-0.28; 0.06]	0.22
	Empagliflozin	-0.74	[-1.09; -0.38]	<0.0001
Erythrocytes	LPC 16:1 SN1	0.10	[-0.09; 0.28]	0.29
	PC 34:5	0.10	[-0.08; 0.27]	0.27
	PE 16:0_18:2	0.10	[-0.07; 0.28]	0.24
	Empagliflozin	0.68	[0.31; 1.04]	0.00032
HbA1c	LPC 16:1 SN1	0.19	[0.01; 0.37]	0.036
	PC 34:5	0.01	[-0.16; 0.18]	0.89
	PE 16:0_18:2	0.23	[0.06; 0.40]	0.0084
	Empagliflozin	0.10	[-0.26; 0.46]	0.58
Hemoglobin	LPC 16:1 SN1	0.02	[-0.16; 0.21]	0.8
	PC 34:5	0.06	[-0.12; 0.23]	0.52
	PE 16:0_18:2	0.12	[-0.06; 0.30]	0.18
	Empagliflozin	0.46	[0.09; 0.83]	0.016
Hematocrit	LPC 16:1 SN1	0.10	[-0.08; 0.29]	0.28
	PC 34:5	0.05	[-0.12; 0.23]	0.55
	PE 16:0_18:2	0.09	[-0.09; 0.26]	0.33
	Empagliflozin	0.62	[0.25; 0.99]	0.0011
MCHC	LPC 16:1 SN1	-0.18	[-0.36; 0.01]	0.061
	PC 34:5	-0.03	[-0.20; 0.15]	0.77
	PE 16:0_18:2	0.04	[-0.14; 0.22]	0.66
	Empagliflozin	-0.43	[-0.80; -0.06]	0.023
Phosphate	LPC 16:1 SN1	-0.04	[-0.22; 0.14]	0.65
	PC 34:5	-0.04	[-0.21; 0.14]	0.67
	PE 16:0_18:2	-0.07	[-0.25; 0.10]	0.41
	Empagliflozin	0.29	[-0.08; 0.65]	0.12
NT-proBNP	LPC 16:1 SN1	-0.06	[-0.24; 0.13]	0.54
	PC 34:5	0.10	[-0.07; 0.27]	0.25
	PE 16:0_18:2	0.11	[-0.07; 0.28]	0.24
	Empagliflozin	-0.34	[-0.70; 0.03]	0.068
Total Proteins	LPC 16:1 SN1	0.01	[-0.18; 0.19]	0.96
	PC 34:5	0.00	[-0.17; 0.17]	0.98
	PE 16:0_18:2	0.05	[-0.12; 0.23]	0.54
	Empagliflozin	0.59	[0.22; 0.95]	0.0017
Uric acid	LPC 16:1 SN1	-0.06	[-0.22; 0.10]	0.48
	PC 34:5	0.08	[-0.07; 0.23]	0.28
	PE 16:0_18:2	-0.02	[-0.17; 0.13]	0.79
	Empagliflozin	-1.00	[-1.31; -0.68]	<0.0001
Urea	LPC 16:1 SN1	0.09	[-0.09; 0.27]	0.31
	PC 34:5	-0.04	[-0.21; 0.13]	0.66
	PE 16:0_18:2	0.05	[-0.12; 0.22]	0.56
	Empagliflozin	0.42	[0.06; 0.78]	0.021
Weight	LPC 16:1 SN1	-0.09	[-0.25; 0.06]	0.23
	PC 34:5	0.15	[0.01; 0.30]	0.039
	PE 16:0_18:2	0.10	[-0.05; 0.24]	0.2
	Empagliflozin	-0.79	[-1.09; -0.48]	<0.0001

Supplementary Figure 4

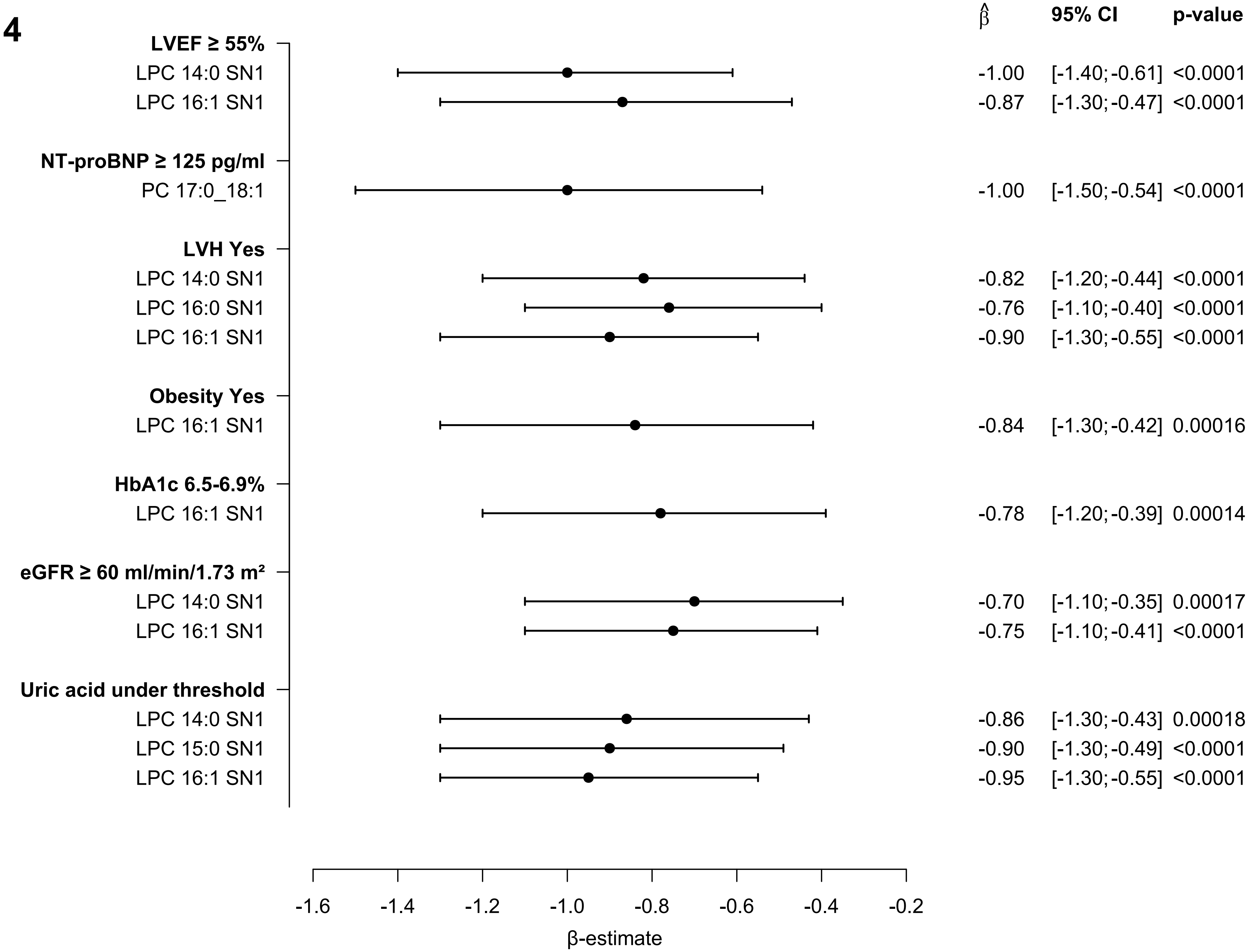

Supplementary Figure 5

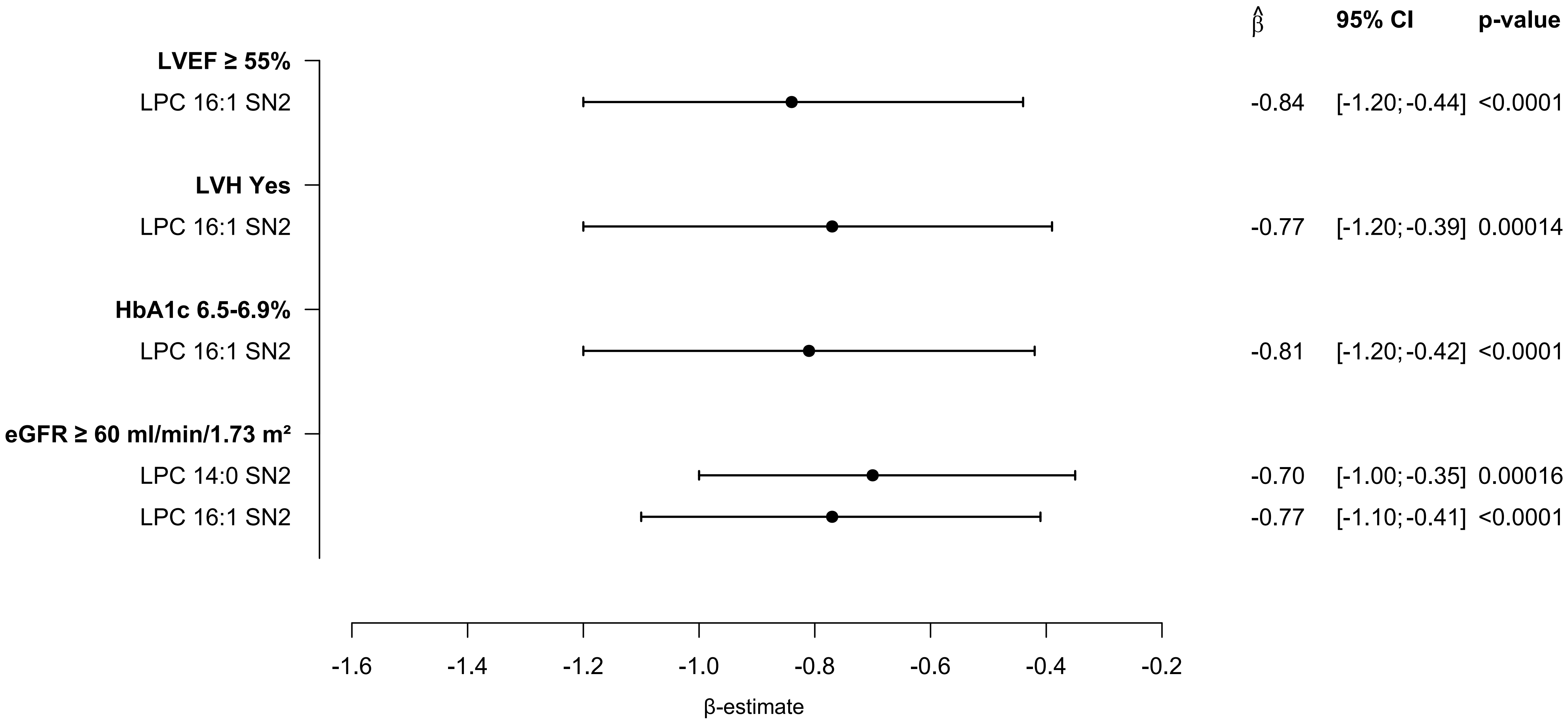

Supplement: Supplementary file 1 [file 12933_2025_2916_MOESM1_ESM.pdf]
