# Supplementary material for: Effect of Empagliflozin on the plasma lipidome in patients with type 2 diabetes mellitus: results from the EmDia clinical trial
Source: Cardiovasc Diabetol. 2025 Sep 8;24:359. doi: 10.1186/s12933-025-02916-0 (PMC12418620; doi:10.1186/s12933-025-02916-0)
Supplement: Supplementary file 2 [file 12933_2025_2916_MOESM2_ESM.pdf]

Supplementary Table 1: Changes in clinical traits associated with empagliflozin treatment after one week.

Table of changes in clinical traits associated with empagliflozin treatment for one week that were significant according to the Benjamini and Hochberg threshold of 0.010. E/E’, elevated left ventricular end-diastolic pressure, EF, ejection fraction, DBP, diastolic blood pressure, BMI, body mass index, BSA, body surface area, eGFR, estimated glomerular filtration rate, HbA1c, glycated hemoglobin, LVH, left ventricular hypertrophy, MCHC, mean corpuscular hemoglobin concentration.

**Clinical traits**	**Estimate [95% CI]**	**p-value**
**Cardiac Biomarkers**		
E/E’	-0.535 [-1.12; 0.046]	0.071
EF	0.424 [-0.770; 1.62]	0.48
NT-proBNP	-72.64 [-148.1; 2.80]	0.059
**Physical traits**		
Weight [kg]	-1.18 [-1.61; -0.747]	<0.0001
BMI [kg/m²]	-0.416 [-0.594; -0.239]	<0.0001
BSA [m²]	-0.011 [-0.016; -0.006]	<0.0001
HbA1c [%]	-0.009 [-0.084; 0.066]	0.81
**Circulation**		
DBP [mmHg]	-3.05 [-5.07; -1.03]	0.0034
**Hematological traits**		
Hematocrit [%]	1.05 [0.441; 1.66]	0.00086
Erythrocytes [/pL]	0.107 [0.041; 0.172]	0.0016
Hemoglobin [g/dL]	0.239 [0.057; 0.420]	0.010
MCHC [g/dL]	-0.276 [-0.518; -0.034]	0.026
**Kidney function**		
Phosphate [mg/dL]	0.178 [0.048; 0.308]	0.0079
Creatinine [mg/dL]	0.073 [0.037; 0.108]	<0.0001
eGFR [ml/min/1.73m²]	-4.97 [-7.24; -2.70]	<0.0001
Urea [mg/dL]	1.60 [0.306; 2.90]	0.016
Uric acid [mg/dL]	-1.01 [-1.28; -0.747]	<0.0001
Albumin [g/L]	1.36 [0.758; 1.95]	<0.0001
Total proteins [g/L]	2.32 [1.29; 3.35]	<0.0001

Supplementary Table 2: Changes in clinical traits associated with empagliflozin treatment after twelve weeks.

Table of changes in clinical traits associated with empagliflozin treatment for twelve weeks that were significant according to the Benjamini and Hochberg threshold of 0.010. E/E’, elevated left ventricular end-diastolic pressure, EF, ejection fraction, DBP, diastolic blood pressure, BMI, body mass index, BSA, body surface area, eGFR, estimated glomerular filtration rate, HbA1c, glycated hemoglobin, LVH, left ventricular hypertrophy, MCHC, mean corpuscular hemoglobin concentration.

**Clinical traits**	**Estimate [95% CI]**	**p-value**
**Cardiac Biomarkers**		
E/E’	-1.12 [-1.72; -0.522]	0.00031

EF	-0.303 [-1.48; 0.870]	0.61
NT-proBNP	9.68 [-50.5; 69.8]	0.75
**Physical traits**		
Weight [kg]	-2.17 [-2.91; -1.43]	<0.0001
BMI [kg/m²]	-0.711 [-0.990; -0.433]	<0.0001
BSA [m²]	-0.021 [-0.029; -0.013]	<0.0001
HbA1c [%]	-0.45 [-0.661; -0.239]	<0.0001
**Circulation**		
DBP [mmHg]	-1.39 [-3.37; 0.600]	0.17
**Hematological traits**		
Hematocrit [%]	2.91 [2.09; 3.73]	<0.0001
Erythrocytes [/pL]	0.289 [0.197; 0.380]	<0.0001
Hemoglobin [g/dL]	0.82 [0.551; 1.09]	<0.0001
MCHC [g/dL]	-0.36 [-0.612; -0.114]	0.0045
**Kidney function**		
Phosphate [mg/dL]	0.123 [0.004; 0.243]	0.043
Creatinine [mg/dL]	0.041 [0.004; 0.078]	0.029
eGFR [ml/min/1.73m²]	-2.98 [-5.48; -0.474]	0.020
Urea [mg/dL]	1.89 [0.585; 3.20]	0.0049
Uric acid [mg/dL]	-0.655 [-1.016; -0.293]	0.00049
Albumin [g/L]	1.24 [0.463; 2.01]	0.0019
Total proteins [g/L]	1.403 [0.110; 2.70]	0.034

Supplementary Table 3: PC1, PC2 and PC3 dimensions of lipid species based on the 37 lipids selected by the sparse group LASSO regularized regression model after treatment for one week.

Table of the 37 lipid species selected by the sparse group LASSO regularized regression model after treatment for one week and the calculated PC1, PC2, and PC3 dimensions.

Lipid species	PC1	PC2	PC3
LPC 14:0 SN1	0.61	.	-0.33
LPC 16:0 SN1	0.77	.	.
LPC 16:1 SN1	0.76	.	.
LPC 18:1 SN1	0.72	.	.
LPC 18:1 SN2	0.68	.	-0.39
LPC 20:2 SN1	0.66	.	-0.35
LPC 20:5 SN1	0.54	.	-0.35
LPC 22:5 SN1	0.67	.	-0.38
PC 15:0_18:2	.	.	.
PC 16:0_20:4	.	.	0.58
PC 17:0_18:1	0.55	.	0.54
PC 18:0_22:4	0.37	.	0.38
PC 18:0_22:6	.	.	0.50
PC 18:1_14:0	0.59	.	0.31
PC 20:0_18:2	.	.	.
PC 33:1	0.62	.	0.41
PC 34:5	0.35	.	.

PC 40:1	.	0.36	.
PC 42:9	0.37	.	0.37
PC O-16:1_22:6	.	.	.
PC O-18:0_18:1	.	0.64	.
PC O-18:0_20:4	.	0.43	0.34
PC O-18:1_20:4	.	.	0.34
PC O-24:1_20:4	.	0.75	.
PC O-35:3	.	.	0.42
PE 16:0_18:2	.	.	.
PE 16:0_20:4	.	.	.
PE 18:0_18:2	.	.	.
PE 18:0_22:6	.	.	0.35
PE 18:2_20:4	.	.	.
PE O-17:1_22:6	.	.	.
SM 34:0;3O	.	0.45	.
SM d18:1_18:1	.	0.67	.
SM d35:1	.	0.79	.
SM d44:2	.	0.73	.
SM d44:3	.	0.80	.
TG 18:2_18:2_18:3	.	.	.

Supplementary table 4: PCA parameters based on the 37 lipids selected by the sparse group LASSO regularized regression model after treatment for one week.

Table of parameters for the PC1, PC2, and PC3 dimensions based on the 37 lipids selected by the sparse group LASSO regularized regression model after twelve weeks of treatment. Parameters include SS loadings, proportion and cumulative variances, proportion explained, and the cumulative proportion.

Lipid species	PC1	PC2	PC3
SS loadings	5.94	4.38	3.15
Proportion Variance	0.16	0.12	0.09
Cumulative Variance	0.16	0.28	0.36
Proportion Explained	0.44	0.33	0.23
Cumulative Proportion	0.44	0.77	1.00

Supplementary Table 5: PC1, PC2 and PC3 dimensions of lipid species based on the 24 lipids selected by the sparse group LASSO regularized regression model after treatment for twelve weeks.

Table of the 24 lipid species selected by the sparse group LASSO regularized regression model after treatment for twelve weeks and the calculated PC1, PC2, and PC3 dimensions.

Lipid species	PC1	PC2	PC3
Cer d18:1_16:0	0.55	0.46	.
Cer d43:1	0.39	0.58	.
Cer d44:1	0.42	0.60	.
LPC 14:0 SN1	0.73	-0.37	-0.32

LPC 14:0 SN2	0.69	-0.51	.
LPC 16:1 SN1	0.70	-0.36	.
LPC 16:1 SN2	0.66	-0.55	.
LPC 20:3 SN1	0.68	.	.
LPC 22:4 SN2	.	-0.42	0.46
PC 14:0_22:6	0.34	.	.
PC 15:0_18:2	0.40	0.42	-0.34
PC 16:0_22:4	.	.	0.56
PC 18:0_22:4	0.69	.	.
PC 34:5	0.50	.	-0.31
PC O-16:1_18:2	.	0.37	.
PC O-22:2_18:2	0.31	0.48	0.49
PC O-24:1_20:4	.	0.34	0.64
PC O-37:5	.	.	.
PE 16:0_18:2	0.42	.	.
PE O-16:1_22:6	.	0.44	.
PE O-20:1_18:2	0.35	0.48	.
SM d18:1_16:1	0.45	.	0.46
SM d42:4	.	.	0.48
TG 16:2_18:2_18:3	0.37	.	.

Supplementary Table 6: PCA parameters based on the 24 lipids selected by the sparse group LASSO regularized regression model after treatment for twelve weeks.

Table of parameters for the PC1, PC2, and PC3 dimensions based on the 24 lipids selected by the sparse group LASSO regularized regression model after one week of treatment. Parameters include the Sum of Squared loadings (SS loadings), as well as proportion and cumulative variances, proportion explained, and the cumulative proportion.

Parameters	PC1	PC2	PC3
SS loadings	5.01	3.34	2.37
Proportion Variance	0.21	0.14	0.10
Cumulative Variance	0.21	0.35	0.45
Proportion Explained	0.47	0.31	0.22
Cumulative Proportion	0.47	0.78	1.00

Supplementary Table 7*:* Multiple linear regression model to predict empagliflozin with lipids after one week of treatment, only results with p-value < 0.05 are shown.

Table of the significant lipids selected by the multiple linear regression model, adjusted for age, sex, and E/E' at baseline to predict empagliflozin with lipids after one week of treatment. Based on the number of singular models, FDR and Bonferroni values were calculated.

**Lipids**	**OR**	**L 95% CI**	**U 95% CI**	**p-value**	**p-value (FDR)**	**p-value (Bonferroni)**	**AUC**

LPC 16:1 SN1	0.061	0.016	0.241	**0.000065**	**0.018**	**0.018**	0.7251
LPC 14:0 SN1	0.112	0.034	0.369	**0.00032**	**0.044**	0.087	0.7003
PC 18:1_14:0	0.110	0.031	0.384	**0.00055**	0.050	0.15	0.6952
PC 33:1	0.096	0.024	0.377	**0.00080**	0.054	0.22	0.6984
LPC 22:5 SN1	0.218	0.082	0.582	**0.0023**	0.13	0.63	0.6936
LPC 14:0 SN2	0.293	0.129	0.667	**0.0034**	0.14	0.92	0.6641
LPC 18:1 SN2	0.000	0.000	0.081	**0.0035**	0.14	0.95	0.6587
LPC 18:1 SN1	0.113	0.025	0.513	**0.0047**	0.14	1.00	0.6612
LPC 20:2 SN1	0.241	0.089	0.655	**0.0052**	0.14	1.00	0.6780
LPC 15:0 SN2	0.252	0.094	0.677	**0.0062**	0.14	1.00	0.6660
LPC 16:0 SN1	0.121	0.027	0.550	**0.0062**	0.14	1.00	0.6845
SM d18:1_18:1	8.681	1.824	41.311	**0.0066**	0.14	1.00	0.6652
LPC 20:5 SN1	0.345	0.159	0.747	**0.0069**	0.14	1.00	0.6581
LPC 20:3 SN1	0.213	0.065	0.692	**0.010**	0.15	1.00	0.6963
PC 17:0_18:1	0.066	0.008	0.523	**0.010**	0.15	1.00	0.6595
LPC 15:0 SN1	0.213	0.064	0.707	**0.011**	0.15	1.00	0.6631
PC O-24:1_20:4	5.660	1.495	21.433	**0.011**	0.15	1.00	0.6570
TG 14:0_16:0_16:0	0.615	0.423	0.895	**0.011**	0.15	1.00	0.6522
PC 18:0_22:4	0.253	0.086	0.744	**0.012**	0.15	1.00	0.6568
SM d35:1	3.803	1.343	10.768	**0.012**	0.15	1.00	0.6530
PC 42:9	0.327	0.135	0.793	**0.013**	0.15	1.00	0.6625
PE O-16:1_22:6	5.990	1.433	25.041	**0.014**	0.15	1.00	0.6765
SM d44:2	363.777	3.314	39925.901	**0.014**	0.15	1.00	0.6677
TG 14:0_16:0_18:1	0.528	0.317	0.879	**0.014**	0.15	1.00	0.6570
LPC 16:0 SN2	0.173	0.042	0.710	**0.015**	0.15	1.00	0.6593
LPC 16:1 SN2	0.470	0.255	0.865	**0.015**	0.15	1.00	0.6711
PC O-18:0_20:4	7.014	1.465	33.578	**0.015**	0.15	1.00	0.6532
TG 13:0_16:1_16:1	0.612	0.412	0.910	**0.015**	0.15	1.00	0.6427
TG 10:0_14:1_16:0	0.722	0.554	0.942	**0.016**	0.15	1.00	0.6570
PE O-18:2_22:6	4.736	1.293	17.348	**0.019**	0.17	1.00	0.6455
SM d44:3	1341.59	3.187	564817.722	**0.019**	0.17	1.00	0.6564
SM d36:3	1746.72	3.045	1002104.77	**0.021**	0.18	1.00	0.6530
PC 34:5	0.396	0.179	0.876	**0.022**	0.18	1.00	0.6515
TG 18:2_18:2_18:3	0.729	0.553	0.962	**0.026**	0.21	1.00	0.6106
TG 14:0_16:0_16:1	0.659	0.454	0.955	**0.028**	0.22	1.00	0.6398
LPE 16:0 SN1	0.200	0.046	0.872	**0.032**	0.24	1.00	0.6431
PC 15:0_20:3	0.242	0.066	0.885	**0.032**	0.24	1.00	0.6394
SM d18:1_24:1	1.086	1.007	1.172	**0.033**	0.24	1.00	0.6333
TG 12:0_16:0_16:1	0.722	0.533	0.977	**0.035**	0.24	1.00	0.6324
SM d18:1_18:0	1.159	1.010	1.330	**0.036**	0.24	1.00	0.6360
TG 16:2_18:2_18:3	0.777	0.614	0.985	**0.037**	0.24	1.00	0.6362
SM d43:2	2.440	1.049	5.675	**0.038**	0.24	1.00	0.6383
TG 14:0_14:0_16:0	0.713	0.518	0.982	**0.038**	0.24	1.00	0.6297
DG 16:0_16:1	0.715	0.520	0.984	**0.040**	0.25	1.00	0.6545
LPE 18:2 SN2	0.480	0.235	0.979	**0.043**	0.25	1.00	0.6219
TG 14:0_15:0_16:1	0.715	0.516	0.990	**0.043**	0.25	1.00	0.6410
TG 10:0_14:0_16:0	0.747	0.562	0.993	**0.044**	0.25	1.00	0.6476
LPE 18:1 SN1	0.296	0.090	0.972	**0.045**	0.25	1.00	0.6331
LPC 22:4 SN1	0.453	0.208	0.987	**0.046**	0.25	1.00	0.6620
PC 18:0_18:1	0.219	0.049	0.983	**0.047**	0.25	1.00	0.6211
PE O-17:1_22:6	23.912	1.007	567.583	**0.049**	0.25	1.00	0.6289
SM 34:0;3O	43.687	1.015	1881.147	**0.049**	0.25	1.00	0.6354

Supplementary Table 8*:* Multiple linear regression model to predict empagliflozin with lipids after twelve weeks of treatment, only results with p-value < 0.05 are shown***.***

Table of the significant lipids selected by the multiple linear regression model, adjusted for age, sex, and E/E' at baseline to predict empagliflozin with lipids after twelve weeks of treatment. Based on the number of singular models, FDR and Bonferroni values were calculated.

**Lipids**	**OR**	**L 95% CI**	**U 95% CI**	**p-value**	**p-value (FDR)**	**p-value (Bonferroni)**	**AUC**
LPC 16:1 SN2	0.104	0.031	0.343	**0.00021**	**0.048**	0.057	0.7154
LPC 16:1 SN1	0.083	0.021	0.324	**0.00035**	**0.048**	0.095	0.7163
LPC 14:0 SN2	0.133	0.042	0.423	**0.00064**	0.058	0.17	0.7070
LPC 14:0 SN1	0.179	0.062	0.517	**0.0015**	0.10	0.41	0.6916
LPC 20:3 SN1	0.156	0.044	0.558	**0.0043**	0.17	1.00	0.6955
LPC 22:4 SN1	0.190	0.061	0.594	**0.0043**	0.17	1.00	0.6896
TG 16:2_18:2_18:3	0.688	0.532	0.890	**0.0044**	0.17	1.00	0.6828
Cer d43:1	4.737	1.558	14.400	**0.0061**	0.19	1.00	0.6835
PC O-22:2_18:2	6.910	1.671	28.574	**0.0076**	0.19	1.00	0.6860
SM d42:4	0.033	0.003	0.405	**0.0076**	0.19	1.00	0.6715
PC 34:5	0.388	0.192	0.783	**0.0082**	0.19	1.00	0.6905
TG 10:0_14:1_16:0	0.720	0.564	0.920	**0.0086**	0.19	1.00	0.6805
Cer d44:1	13.635	1.748	106.37	**0.013**	0.25	1.00	0.6808
PC O-24:1_20:4	4.125	1.346	12.645	**0.013**	0.25	1.00	0.6726
LPC 18:1 SN2	0.000	0.000	0.208	**0.015**	0.27	1.00	0.6654
LPC 20:5 SN2	0.510	0.294	0.886	**0.017**	0.27	1.00	0.6611
PE O-20:1_18:2	85.513	2.205	3316.95	**0.017**	0.27	1.00	0.6740
LPC 20:3 SN2	0.406	0.190	0.867	**0.020**	0.30	1.00	0.6557
TG 16:1_18:2_18:3	0.677	0.482	0.952	**0.025**	0.35	1.00	0.6590
LPC 22:4 SN2	0.541	0.314	0.933	**0.027**	0.35	1.00	0.6489
PE O-16:1_22:6	4.445	1.165	16.953	**0.029**	0.35	1.00	0.6609
Cer d18:1_24:0	1.376	1.029	1.841	**0.031**	0.35	1.00	0.6710
LPC 16:0 SN1	0.155	0.028	0.843	**0.031**	0.35	1.00	0.6622
TG 14:0_16:0_18:1	0.639	0.426	0.959	**0.031**	0.35	1.00	0.6622
LPC 20:5 SN1	0.439	0.200	0.961	**0.039**	0.37	1.00	0.6636
TG 14:0_16:0_16:1	0.724	0.533	0.983	**0.039**	0.37	1.00	0.6505
TG 15:0_16:1_16:1	0.708	0.510	0.983	**0.039**	0.37	1.00	0.6518
TG 12:0_16:1_18:1	0.730	0.541	0.986	**0.040**	0.37	1.00	0.6532
TG 14:0_16:1_18:1	0.679	0.469	0.983	**0.040**	0.37	1.00	0.6480
LPC 20:4 SN2	0.518	0.276	0.974	**0.041**	0.37	1.00	0.6410
LPC 15:0 SN2	0.360	0.132	0.979	**0.045**	0.37	1.00	0.6534
PC 18:0_22:4	0.385	0.150	0.989	**0.047**	0.37	1.00	0.6665
PC 18:1_14:0	0.426	0.184	0.990	**0.047**	0.37	1.00	0.6529
TG 14:0_16:0_16:0	0.755	0.572	0.997	**0.048**	0.37	1.00	0.6570

Supplementary Table 9: Association between clinical traits and lipids within the placebo group after 12 weeks of treatment

Lipids with consistent Empagliflozin-driven change in abundance among timepoints were used as covariates in a model in which changes in clinical traits between baseline and twelve weeks within individuals in the placebo group were used as outcome.

Clinical trait	Lipid	Coefficient for lipid in sparse group LASSO model, α = ⅓	Estimate	p-value	Group
**Cardiac function and biomarkers**					
NT-proBNP	LPC 16:1 SN1	-0.051	-123.747 [-235.345; -12.148]	0.03	Placebo
**Renal function**					
Creatinine	PC 34:5	-0.026	-0.091 [-0.143; -0.04]	0.00076	Placebo
eGFR	PC 34:5	-0.026	5.808 [2.26; 9.357]	0.0017	Placebo
Urea	PE 16:0_18:2	0.012	-3.301 [-6.161; -0.44]	0.024	Placebo
**Physiological and circulatory health**					
Hemoglobin	LPC 16:1 SN1	-0.051	0.885 [0.23; 1.54]	0.0089	Placebo
Hematocrit	LPC 16:1 SN1	-0.051	2.206 [0.213; 4.198]	0.031	Placebo
DBP	PE 16:0_18:2	0.012	5.611 [0.357; 10.864]	0.037	Placebo
DBP	LPC 16:1 SN1	-0.051	5.56 [0.292; 10.827]	0.039	Placebo

Supplementary Table 10: Summary table of clinical trait-lipid associations based on singular linear regression models per clinical trait (Bonferroni-correction with a threshold of 0.000183 was used) after one week of empagliflozin treatment.

Table of lipids that are significantly associated (Bonferroni-threshold of 0.000183 was used) with the selected clinical traits after one week of treatment with empagliflozin.

Clinical trait	Lipid	Beta-estimate	p-value
**Erythrocytes**	**PE 18:0_22:6**	**0.065**	**0.00016**
Hematocrit	PE 18:0_22:6	0.544	0.00079
eGFR	PC O-18:0_20:4	-1.96	0.0015
Uric acid	PC 42:9	0.245	0.0016
Total proteins	PC O-18:0_20:4	0.863	0.002
Weight	SM d18:1_18:1	-0.334	0.002
BMI	LPC 14:0 SN1	0.138	0.0021
DBP	PC O-18:0_20:4	-1.54	0.0023

Albumin	PC O-18:0_20:4	0.496	0.0024
BSA	PC O-18:0_20:4	-0.00393	0.0027
Weight	LPC 14:0 SN1	0.317	0.0032
Erythrocytes	PE 18:0_22:6	0.0506	0.0034
Erythrocytes	SM d18:1_18:1	0.051	0.0036
Creatinine	PC O-18:0_20:4	0.0279	0.004
Hematocrit	SM d18:1_18:1	0.461	0.0041
Uric acid	PC O-24:1_20:4	-0.222	0.0043
Albumin	SM d18:1_18:1	0.464	0.0043
Total proteins	SM d18:1_18:1	0.772	0.0057
Hemoglobin	SM d18:1_18:1	0.131	0.0059
DBP	LPC 18:1 SN2	1.33	0.0082
Erythrocytes	PC O-18:0_18:1	0.0458	0.0088
Erythrocytes	PC O-18:0_20:4	0.0453	0.0093
Hematocrit	PC O-18:0_18:1	0.426	0.0095
Erythrocytes	SM d35:1	0.0446	0.01
Total proteins	PC O-24:1_20:4	0.707	0.011
Weight	PC O-18:0_20:4	-0.278	0.011
Hemoglobin	PE 18:0_22:6	0.12	0.013
Hematocrit	PC O-18:0_20:4	0.406	0.013
Total proteins	SM d44:2	0.701	0.013
Erythrocytes	PC O-24:1_20:4	0.0427	0.015
Hemoglobin	PC O-18:0_20:4	0.117	0.015
Weight	PC 34:5	0.267	0.015
Hematocrit	PC O-24:1_20:4	0.392	0.016
Total proteins	SM d35:1	0.676	0.016
Weight	SM d35:1	-0.265	0.016
Total proteins	SM d44:3	0.663	0.018
Albumin	PC O-24:1_20:4	0.382	0.019
Weight	PC 18:1_14:0	0.26	0.019
Weight	PC O-24:1_20:4	-0.256	0.019

Hematocrit	SM d44:2	0.381	0.02
Hematocrit	SM d35:1	0.371	0.023
Uric acid	PC O-18:0_18:1	-0.182	0.023
Albumin	LPC 20:5 SN1	-0.379	0.023
Weight	PC 42:9	0.246	0.024
Uric acid	PC 34:5	0.179	0.025
Albumin	SM d44:2	0.368	0.025
eGFR	PC O-24:1_20:4	-1.37	0.027
Phosphate	LPC 20:5 SN1	-0.0715	0.03
Uric acid	SM d35:1	-0.167	0.037
Hemoglobin	PC O-18:0_18:1	0.101	0.039
Creatinine	PE 18:0_18:2	0.0203	0.039
Total proteins	PC O-16:1_22:6	0.578	0.041
Albumin	LPC 14:0 SN1	-0.331	0.042
Weight	PC O-18:0_18:1	-0.226	0.042
Weight	SM 34:0;3O	-0.222	0.046
Creatinine	PC O-24:1_20:4	0.0191	0.048

Supplementary Table 11: Summary table of clinical trait-lipid associations based on singular linear regression models per clinical trait (Bonferroni-correction with a threshold of 0.000183 was used) after twelve weeks of empagliflozin treatment.

Table of lipids that are significantly associated (Bonferroni-threshold of 0.000183 was used) with the selected clinical traits after twelve weeks of treatment with empagliflozin.

**Clinical trait**	**Lipid**	**Beta-estimate**	**p-value**
**Weight**	**LPC 14:0 SN1**	**0.73**	**0.000016**
**Weight**	**LPC 16:1 SN1**	**0.716**	**0.00002**
**BSA**	**LPC 14:0 SN1**	**0.00752**	**0.000065**
**BMI**	**LPC 16:1 SN1**	**0.233**	**0.00013**
Weight	LPC 16:1 SN2	0.63	0.00027
BSA	LPC 16:1 SN1	0.00678	0.00031
Weight	LPC 14:0 SN2	0.607	0.00045
BMI	LPC 16:1 SN1	0.233	0.00068
BSA	PC O-24:1_20:4	-0.00619	0.0013
BMI	LPC 14:0 SN1	0.21	0.0014
BSA	LPC 16:1 SN2	0.00616	0.0014
E/E'	PC O-24:1_20:4	-0.49	0.0015
BMI	LPC 14:0 SN2	0.199	0.0016

Weight	PC 18:0_22:4	0.549	0.0016
Weight	LPC 20:3 SN1	0.559	0.0018
Hemoglobin	Cer d44:1	0.237	0.0019
Weight	PC O-24:1_20:4	-0.545	0.0019
BSA	LPC 20:3 SN1	0.00598	0.0024
Hematocrit	PE O-16:1_22:6	0.719	0.0026
BSA	LPC 14:0 SN2	0.0057	0.0032
Weight	PC O-37:5	-0.509	0.0035
BSA	PC 18:0_22:4	0.00561	0.0036
Albumin	Cer d43:1	0.574	0.0039
Albumin	Cer d44:1	0.566	0.0045
BMI	LPC 16:1 SN2	0.197	0.0052
BSA	PC O-37:5	-0.00535	0.0053
Hemoglobin	Cer d43:1	0.214	0.0055
Hematocrit	Cer d44:1	0.659	0.0057
MCHC	SM d18:1_16:1	0.177	0.0069
MCHC	LPC 16:1 SN1	0.17	0.0074
HbA1c	LPC 16:1 SN1	0.148	0.0082
Hemoglobin	PC O-22:2_18:2	0.206	0.0083
Albumin	Cer d18:1_16:0	0.523	0.0092
Erythrocytes	PC O-22:2_18:2	0.0681	0.0095
Hematocrit	Cer d43:1	0.622	0.01
HbA1c	LPC 16:1 SN2	0.145	0.01
Urea	PC 14:0_22:6	-0.876	0.01
Weight	PC 34:5	0.448	0.011
Hemoglobin	Cer d18:1_16:0	0.192	0.013
Hemoglobin	PE O-16:1_22:6	0.194	0.013
Erythrocytes	PE O-16:1_22:6	0.0649	0.013
MCHC	PC 18:0_22:4	0.16	0.013
MCHC	TG 16:2_18:2_18:3	0.162	0.013
BMI	PC 34:5	0.175	0.014
BMI	LPC 20:3 SN1	0.159	0.014
Hemoglobin	PC O-16:1_18:2	0.193	0.014
Albumin	PC O-24:1_20:4	0.492	0.015
Urea	PC O-37:5	0.808	0.016
Hematocrit	PC O-16:1_18:2	0.583	0.018
Hemoglobin	PE O-20:1_18:2	0.184	0.019
MCHC	LPC 20:3 SN1	0.153	0.019
Hematocrit	PC O-22:2_18:2	0.562	0.021
E/E'	PE O-20:1_18:2	-0.362	0.021
BMI	PC 18:0_22:4	0.156	0.023
Erythrocytes	Cer d44:1	0.0594	0.023
Urea	LPC 14:0 SN1	-0.767	0.023
BMI	PC 15:0_18:2	-0.145	0.024
Weight	PC O-22:2_18:2	-0.396	0.026

Weight	TG 16:2_18:2_18:3	0.399	0.026
MCHC	PE 16:0_18:2	0.142	0.027
BMI	PC O-37:5	-0.143	0.028
Urea	TG 16:2_18:2_18:3	-0.743	0.029
MCHC	LPC 16:1 SN2	0.141	0.029
Weight	PC 15:0_18:2	-0.385	0.031
Hemoglobin	PC 15:0_18:2	0.17	0.033
Hematocrit	PE O-20:1_18:2	0.516	0.035
BMI	PC O-24:1_20:4	-0.14	0.036
Erythrocytes	Cer d43:1	0.055	0.036
Urea	PC 34:5	-0.707	0.037
HbA1c	PC 18:0_22:4	0.118	0.039
Hematocrit	TG 16:2_18:2_18:3	-0.506	0.04
BSA	PC O-22:2_18:2	-0.00402	0.041
MCHC	LPC 14:0 SN1	0.132	0.041
Urea	PC O-16:1_18:2	0.694	0.042
BSA	TG 16:2_18:2_18:3	0.00399	0.044
E/E'	LPC 14:0 SN2	0.316	0.045
HbA1c	LPC 14:0 SN2	0.113	0.048
