# Supplementary material for: Effect of Empagliflozin on the plasma lipidome in patients with type 2 diabetes mellitus: results from the EmDia clinical trial
Source: Cardiovasc Diabetol. 2025 Sep 8;24:359. doi: 10.1186/s12933-025-02916-0 (PMC12418620; doi:10.1186/s12933-025-02916-0)
Supplement: Supplementary file 3 [file 12933_2025_2916_MOESM3_ESM.pdf]

Supplementary Figure 1: Principal Component Analysis (PCA) at baseline for empagliflozin- and placebo-treated individuals based on the lipid-signatures after one and twelve weeks respectively
A PCA based on the 37 selected lipids by the sparse group LASSO regularized regression model after treatment for one week
B PCA based on the 24 selected lipids by the sparse group LASSO regularized regression model after treatment for twelve weeks

Supplementary Figure 2: Receiver operating characteristics (ROC) analysis for the selected lipid-signatures after one and twelve weeks respectively
A ROC for the 37 selected lipids by the sparse group LASSO regularized regression model after treatment for one week with an AUC of 0.923, a sensitivity of 0.797 and a specificity of 0.899.
B ROC for the 24 selected lipids by the sparse group LASSO regularized regression model after treatment for twelve weeks with an AUC of 0.864, a sensitivity of 0.723 and a specificity of 0.794.

Supplementary Figure 3: Forest-plot of the association of individual lipids with selected subgroups after empagliflozin treatment for one week adjusted by sex, age and E/E' at baseline).
Using linear regression with Bonferroni-correction (threshold = 0.000183), a forest plot was generated to show the association of individual lipids with selected clinical subgroups after empagliflozin treatment for one week.

Supplementary Figure 4: Forest-plot of the association of individual lipids with selected subgroups after empagliflozin treatment for one week.
Using linear regression with Bonferroni-correction (threshold = 0.000183), a forest plot was generated to show the association of individual lipids with selected clinical subgroups after empagliflozin treatment for one week.

Supplementary Figure 5: Forest-plot of the association of individual lipids with selected subgroups after empagliflozin treatment for twelve weeks.
Using linear regression with Bonferroni-correction (threshold = 0.000183), a forest plot was generated to show the association of individual lipids with selected clinical subgroups after empagliflozin treatment for twelve weeks.
